# Effect of climate change on distribution of species of common horned frogs in South America

**DOI:** 10.1371/journal.pone.0202813

**Published:** 2018-09-12

**Authors:** Kleber Silva Vieira, Paulo Fernandes Guedes Montenegro, Gindomar Gomes Santana, Washington Luiz da Silva Vieira

**Affiliations:** 1 Laboratório de Ecofisiologia Animal do Departamento de Sistemática e Ecologia da Universidade Federal da Paraíba, João Pessoa, Paraíba; Brasil; 2 Bolsista CNPq pelo Programa de Desenvolvimento Científico e Tecnológico Regional (FAPESQPB)/Universidade Estadual da Paraíba, Campina Grande, Paraíba; Brasil; 3 Bolsista do Programa Nacional de Pós-Doutorado (PNPD), Programa de Pós-Graduação em Ecologia e Conservação (PPGEC)/CCBS, Universidade Estadual da Paraíba, Campina Grande, Paraíba; Brasil; Universitat Trier, GERMANY

## Abstract

Our main objectives were to verify the effect of climate change on distribution of frogs of the family Ceratophryidae and if the legal protection areas in South America will be effective or ineffective in ensuring the preservation of the toads this family in coming decades. The results showed that in the last 140,000 years, species of the family Ceratophryidae expanded and contracted their distribution areas, which naturally reflected the climate and vegetation changes in the Quaternary of South America. The maps of projections showed that changes in temperature determined the area of habitat suitability of 63.7% of the species of ceratophrids both during the last interglacial period and nowadays, and it seems that this will also be the case for the next 62 years. Given the current concerns about future extinctions in the tropics, it is prudent to examine, with special attention, the effects of climate fluctuations on the diversity and distribution of species, because the current estimates of reduction in biodiversity caused by habitat destruction and emission of greenhouse gases are comparable to estimated reductions during glacial intervals.

## Introduction

The distribution of a species is, virtually, the geographical area where it is found, or where there is some probability of it being found [1, 2]. A comprehensive definition suggests the distribution of a species as maying be its habitat. This concept is often loosely applied or even confused with the one defining ecological niche because of the close relationship between niche and habitat [3]. The word niche does not suggest a place, but an idea. Niche is regards the limits placed on organisms by environmental conditions. Thus, didactically speaking, a habitat can provide many different niches, where different organisms live, while a niche refers to manner how they live in areas they inhabit [4–6].

Having knowledge of the distribution and the niche of a species is important because such information is critical in ecological research and, without a doubt, for effective methods for biological conservation. Ignorance of the distribution of a species prevents us from knowing their history or what resources it uses, or how variations in birth rate, mortality and migration affect certain populations. It also prevents us from knowing the action and the effect of intra- and interspecific interactions on it, and of the environment as well.

Although known for nearly three hundred years, our knowledge and comprehension about the geographical distribution of species of the family Ceratophryidae leaves much to be desired [7, 8]. What we know about them is restricted to sparse information on their feeding habits, reproduction and development of a few species [9–14]. Such lack of knowledge is directly due in part to their cryptic fossorial habits [8, 15, 16], making it difficult to study adequately the ecological role they play.

For decades the biome was indicated as the determinant in the distribution of some species of ceratophrids [8], suggesting a historical dependence and co-evolutionary with the environments in which they are found, making its geographical distribution restricted to certain areas. If this is true, urgent preservation measures need to be taken by creating more parks and reserves. Such measures are appropriate because according to the IUCN data, destruction of habitat by agriculture, livestock, pollution, deforestation, international animal trafficking and human occupation are important factors in the decline of the Ceratophryidae throughout South America [17].

Although desirable, knowing the exact distribution of a species is impractical and unrealistic. Timed collections, while effective, are lengthy and burdensome, especially in times of ecological crisis where environmental habitats are at serious risk of disappearing in relatively short time. Therefore, the use and application of ecological niche modeling (ENMs) are justifiable, especially because of their ability to generate habitat suitability areas hypotheses based on the probable relationship between certain environmental variables and the occurrence sites of species studied [18] in a short time compared to conducting timed collections.

Despite the data accumulated over years, the distribution of species of the Ceratophryidae is still debatable. Available distribution information is almost always scarce and with gaps for some species [19, 20], indicating that more collection effort would be needed to fill the gaps and thus make it possible to generate better data in modeling studies.

Based on the geographical distribution of ceratophrids currently available, we verified if the protected areas in South America were effective at holding the distribution of the territory of this amphibian species, especially because the anthropic impact in its environmental. Furthermore, we sought to know the habitat suitability area of family representatives and how and why this area changed across the time. We also seek evidences about the effect of global warming on species distribution in the coming decades and what can be done to mitigate or perhaps avoid any possible reduction in the area of distribution of these animals in the near future.

## Materials and methods

All information on the distribution of the species studied was obtained from specimens preserved in scientific collections (presence data). The data came from, in this case, the following sources: information of specimens examined contained on labels and/or records of museum collections, as well as scientific articles (S1 and S2 Tables). For specimens that did not have precise site or geographic coordinates in their records, that information was obtained from the city and/or district near the collection site, up to 10 km. This was make to accommodate spatial inaccuracies in the species occurrences, without causing profound distortions on the data of geographic distribution, since the species studied here are of restricted vagility. Such information was found through the GEOlocate web application [21].

The maps of potential distribution of past, present and future of species of the family Ceratophryidae (S1–S11 Figs) were generated through the DIVA-GIS software—Bioclim [22] and MaxEnt [23], performing it run for each species separately. The MaxEnt use both pseudo absence and presence data randomly sampled from the calibration area and the Bioclim is an envelope-mode method that depict sites that are located within the geographical space potentially occupied by a species [24, 25].

We chose to use the DIVA-GIS and MaxEnt software because they are at the same time simpler and precise for the particularities the species studied here (restricted dispersal properties and niche known, although limited available data), without sacrificing your graphical output nor consistence of the predictions, especially MaxEnt [24, 26], compared to other available software when in specific circumstances [27], principally for species with low vagility and dispersion capacity restricted, as amphibians are when compared to others vertebrates species such several types birds and mammals.

In our study the climatic data of the past, present and future are continuous variables that are part of GIS layers obtained from the WorldClim portal [28]. They were derived from the monthly temperature and rainfall values in order to generate more biologically meaningful variables. In our study, the bioclimatic variables represent annual trends (e.g., mean annual temperature, annual precipitation) seasonality (e.g., annual range in temperature and precipitation) and extreme or limiting environmental factors (e.g., temperature of the coldest and warmest month, and precipitation of the wet and dry quarters).

The layers representing current climates are in a resolution of 2.5 arc-minutes (~4.5 km^2^ at the equator), which contained bioclimatic data from the 1950s to the present. To predict the distribution of species of Ceratophryidae in future climates, we used climate models with a resolution of 30 arc-seconds (0.93 x 0.93 = 0.86 km^2^ at the equator) of the Canadian Centre for Climate Modelling and Analysis–CCCMA (CGCM4/CMIP5). Emission scenarios used were A2 and B2 [29, 30]. This was done to predict the distributions in reasonably optimistic (B2) and/or pessimistic (A2) scenarios of global climate change according to the Intergovernmental Panel on Climate Change—IPCC [31, 32].

The distribution study of the species of Ceratophryidae in paleoclimatic conditions was conducted in the GIS layers of the Last interglacial (~ 120 000–140 000 BP) and the Last glacial maximum (~ 21 000 years BP). The first layer is in a resolution of 30 arc-seconds [33], the other at 2.5 arc-minutes. The latter was generated by the Paleoclimate Modelling Intercomparison Project Phase II (PMIP2). The original data was made available by CMIP5, being downscaled and calibrated (bias corrected) using WorldClim 1.4 as baseline 'current' climate. We performed this analysis in attempt to verify how the area of habitat suitability of ceratophrids probably changed (shifted, widened or decreased) over time. This information is of great importance for large-scale conservation plans, since it is predictive of key regions where species are likely to suffer the effects of habitat destruction [34]. See supporting information to more details (S1 File).

To discriminate the individual percentage contribution of the model (regularized training gain), we applied the Jackknife test to the model in all environmental layers and also generated predictive contribution tables for the variables (S12–S22 Figs). These tables were constructed because the particular predictive power was easily checked for each distribution variable of the species studied [35].

The performance of each estimated model was generated by the "area under curve" (AUC). In this case, AUC values equal or close to 1 indicate excellent accuracy. Values equal to or less than 0.5 are predictive results that are not as good as those obtained randomly [36]. Some studies suggest that the application of ROC AUC is essentially a very reliable measure of accuracy when compared to other estimators [37, 38].

To avoid ambiguity and a wide range of settings [1], we decided to apply the term areapause to spatial limits (outlines) of the suitable area of the species. The proposal of this new term, inspired in the astronomical term heliopause, suggests that the distribution is stopped because of some ecological pressure and because the species is no longer able to adjust to certain areas of the geographical space. Areapause is a virtual term to the most outer border of the distribution (varying according of method and descriptors). Different factors can determine the spatial limit of the distribution of a species. In our study, it was shown by potential distribution, indicted by abiotic factors, here designed through of climatic variables—Grinnellian niche [39, 40].

The rasters containing the models of the potential distribution areas of ceratophrids have also been overlapping to polygons of geographical areas which delimited environmental preservation/conservation areas in South America [41]. This was done to determine how the effective distribution of these frogs was or wasn’t inside in areas protected by law, facilitating in this way, future categorization of the conservation status [42].

## Results

The niche ecological models produced for eleven species of ceratophrids—across subsets of environmental variables of the past, present and future—resulted in a total of 55 predictions (more 33 maps of ecological reserves, whose areas were geometrically extracted of the input features). In the ‘distributions’ generated, the area potentially occupied by species (during some moments in history) showed some interesting variations throughout in time and of the geographical space.

**Fig 1:**
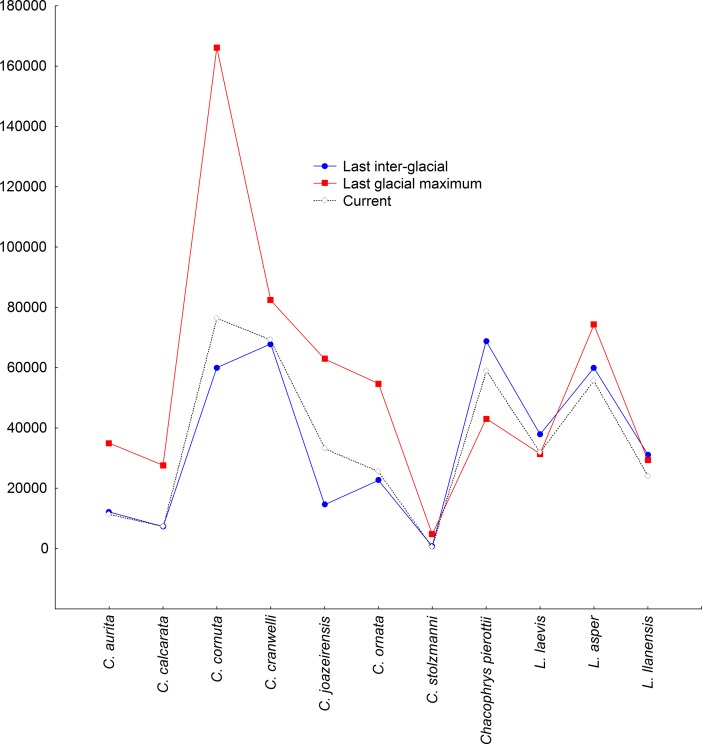
The dynamics of the retraction and expansion of the areas of habitat suitability (km^2^) of Ceratophryidae species during the last 140,000 years.

Our models demonstrated that the horned frogs *C*. *joazeirensis*; *C*. *ornata* and *C*. *stolzmanni* had their smallest recorded areas of habitat suitability during the last inter-glacial, and that their areas varied very little during the climate changes that occurred during that period as compared to the other species of the Ceratophyidae, which demonstrated accentuated contractions or expansions of their areas of habitat suitability projected (Fig 1).

Our results also demonstrated that during the last glacial maximum there was a significant increase in the areas of habitat suitability of almost all of the species we studied, except for *C*. *stolzmanni*, *Chacophrys pierotti*, *L*. *laevis*, and *L*. *llanensis* (whose probability of presence seems larger under more warmer climate), which, despite the low sampling, can be associated for variations in temperature and regime of rains, modulate by South America Monsoon System during this period, which changed the structuration of biomes, mainly in central region of South America, in approximately 18,000 years ago, especially in the Chaco, which coincided with the greatest extensions of the polar ice caps [43].

**Fig 2:**
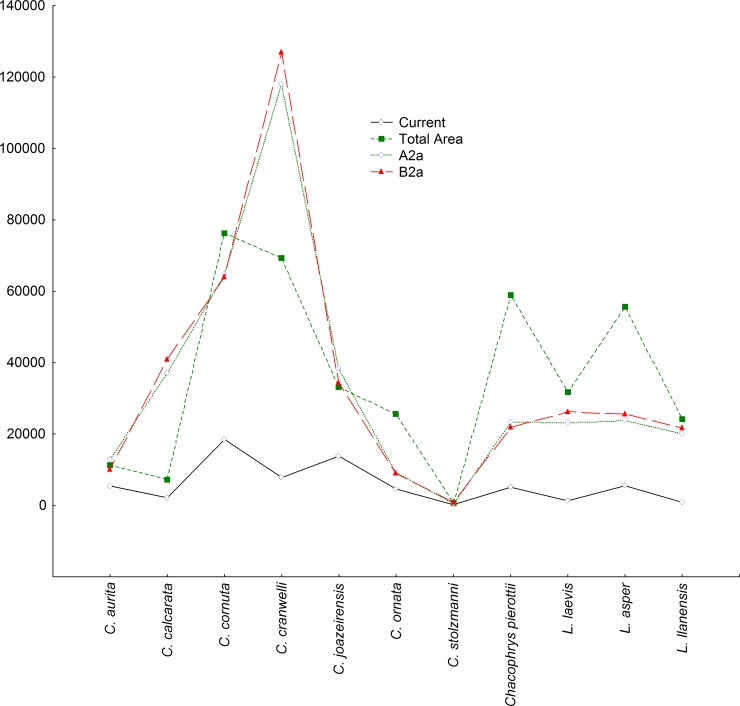
Probable retraction and expansion dynamics of the areas of habitat suitability (km^2^) of Ceratophryidae species within existing legally protected areas until the year 2080, as compared to their current distributions (total and inside protected areas). Data obtained from two projections of CO2 emissions (A2a and B2a).

**Fig 3:**
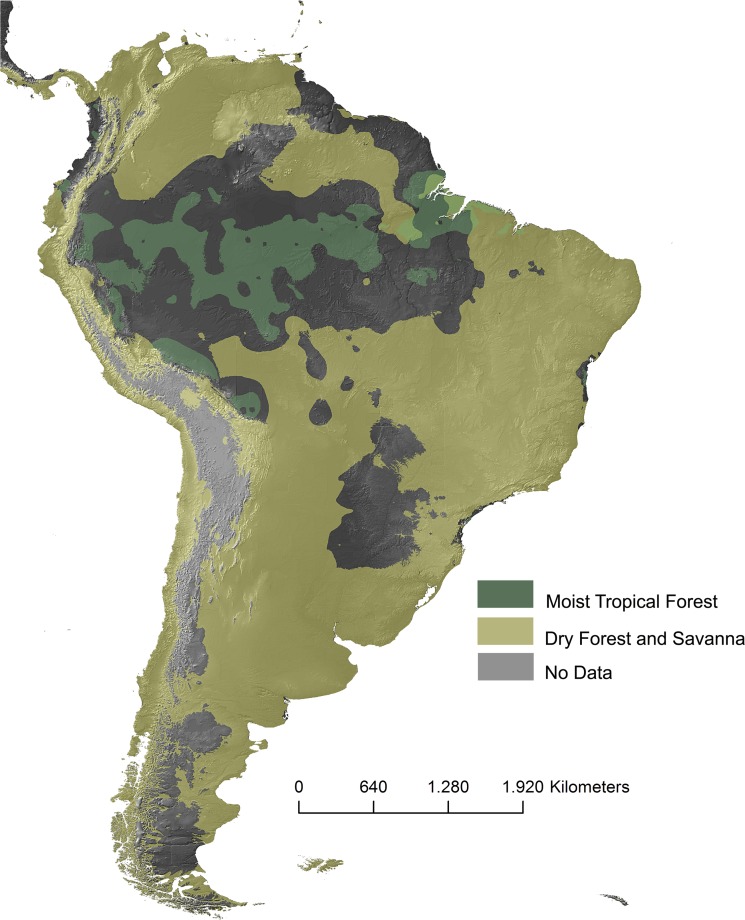
Projected vegetation map of South America in 2080 elaborated through the sum of polygons of the areas of habitat suitability. Data obtained from models generated for Ceratophryidae species based on two projections of CO2 emissions (A2a and B2a).

Curiously, all of the models predicted expansions of the areas occupied by those horned frog species (with the exception of *C*. *stolzmanni*) within ecological reserve areas in approaching decades (Fig 2). Those models likewise suggest that the expansions of those species will occur in response to the gradual intensification and expansion of semi-arid environments (and alteration in regime of rains) in South America until 2080 (Fig 3).

Different from the other Ceratophryidae species, *C*. *crawelli* and *C*. *calcarata* are expected to demonstrate hyper-expansions of their areas of habitat suitability in the next 62 years that will greatly exceed their present distributions–extending even further than they did during the last glacial maximum. That predicted hyper-expansion is explained in our model by variations in minimum temperatures during the coldest and driest months.

## Discussion

First, we must point out something important about our results. The data are treated here in a superficial time plan, that is, in a relatively short period of time. This means that comparisons between the effects of climate change in South America since the Last interglacial and up to the year 2080 are not as absurd or disparate as they may seem, because the species treated here are the link between these two historical moments and can be easily inserted into the current diversity and biogeography as in the Quaternary without major problems [44, 45]. Thus, we can say that the ceratophrids first expanding and then contracting their distribution area in the last 140 000 years, which will naturally occur in the coming decades according to predictions of our models.

The Earth's climate, consequently its biosphere, has undergone profound changes due to the Pleistocene glaciations that occurred over the last few thousand years. Given the current concerns about future extinctions in the tropics, it is prudent to examine with special attention the effects of climate fluctuations on the diversity and distribution of species, both in the past and especially in the future, because the decrease in current estimates of biodiversity, caused by habitat destruction and emission of greenhouse gases, are comparable to estimated reductions during glacial intervals [45]. Therefore, this would justify making a parallel between the likely effects of current climate change on biodiversity with those that occurred during the Quaternary without running the risk of making erroneous observations and arriving at false conclusions.

The distribution of organisms is a multidimensional and multivariate event, and therefore, the relationship between climate and species distribution is not always direct [46]. In addition to climatic factors, interaction processes and resource utilization (Eltonian niche–scenopoetic dimensions) also contribute in determining the limits (areapauses) of the distribution of species [47–49]. Because it is not so simple to discriminate these components, much caution is necessary in the use of projections to indicate a land mass as an effective occupation area of a particular species, either in the past or in the present [50]. Otherwise, there is a risk of making mistakes in interpreting the results. Having secured this, our models showed fluctuations in precipitation affecting the area of habitat suitability of almost all species of ceratophrids during the Last interglacial, remaining an important variable, less in present day, and it seems that it will also be in the coming decades. In contrast, we found that temperature had effected on these toads regarding ‘occupation area’ during the Last glacial maximum, where we found a greater territorial expansion compared to today and the Last interglacial.

In the case of species of ceratophrids, the way these amphibians reacted to climate changes during the last 140 000 years was something very curious in spite of microhabitats where they usually live [10, 12, 51]. We observed that representatives of the genera *Chacophrys* and *Lepidobatarchus* (except *L*. *asper*) had higher area of habitat suitability during the Last interglacial unlike species of *Ceratophrys*—with wider area of habitat suitability during the Last glacial maximum. This suggests that ceratophrids, even though they share many traits, did not respond equally to climate fluctuations, and some species were much more sensitive to the oscillatory effects of temperature (e.g., *C*. *calcarata; C*. *cranwelli*; *C*. *ornata*; *Chacophrys; L*. *asper and L*. *laevis*) while others to precipitation (e.g., *C*. *aurita; C*. *cornuta*; *C*. *joazeirensis; C*. *stolzmanni* and *L*. *llanensis*). Note that it is difficult to make any conclusions about why such conditions affect the species in this way. To further investigate the prospects of ceratophrids under climate change, would be required do physiological studies to investigate the species’ tolerance in different ecological conditions.

The Quaternary was characterized by fairly rapid climate fluctuations, where it affect the distribution of faunas and floras, extinguished many species (mainly mammals and birds) and gave rise to many others in less than a million years [46, 52–54]. For ceratophrids, it is possible that they were already well adapted when they survived the changes of the Quaternary, responding to them with few extinctions and following the dynamics of biomes with which they remained associated and seemingly dependent on for millennia [8, 44, 55]. Like other species, the way these frogs responded to climate change in the last thousands of years defined and still influences their current distributions [8, 56].

Quaternary deposits of South America indicate climate fluctuations to wetter conditions interspersed with drier climates in large areas [56, 57]. This oscillation was responsible for the processes of formation of new types of soil and topography, expanding and retracting forested areas or savanna steppe areas as sculpted microhabitats and new landscapes [56, 58]. The faunas and floras that negatively responded to this dynamics became extinct or relegated to a few scattered refuges [50, 59–61]. In the latter case, we can locate almost all ceratophrids that in response to Quaternary climatic fluctuations increased their area of habitat suitability (probably the effective occupation area) in the glacial phase, something very well represented in our projections. During the Last glacial maximum, the temperature was milder and the dry areas much larger. In contrast, during the wet stages (interglacial), we observed shrinkage of the distribution areas of these species of frogs, which indicates a higher relation of these amphibians to the steppe grasslands than rainforests.

Many transformations occurred in the paleovegetation of South America during glacial periods to interglacial and vice versa, with faunas and floras receding and expanding their ranges over time as a result of global climate change. For the Ceratophryidae, this was no different and necessarily such cycles (retraction and expansion) can will happen again, with possible worsening because of the result of human activity in this century, an at a much shorter period of time than that observed during the Quaternary, something that will be greatly detrimental to the survival of ceratophrids and that also can cause extinction of others species, an event comparable only those that have already occurred during the Permo-Triassic and Cretaceous-Tertiary [62–65].

While we can look at the past and imagine detailed scenarios, it is difficult to predict how ceratophrids, and also many other amphibians [66], will react to environmental change and the gradual increase in temperature as a result of current climate change—probably aggravated by humans. During the glacial phase, the temperature was milder compared to interglacial periods and in these two stages, there was no environmental degradation on a scale as fast, deep and broad as currently observed. This, by itself, unlike today, already provided a good chance of survival to many animal and plant species. Thus, based on this perspective, it may be that the possible expansion of distribution areas proposed by our models is false positive indicators of the future of ceratophrids instead of representing truly optimistic scenarios for them. On this account, it may be that those new areas cannot be colonized by these amphibians. For example, while a cougar can walk kilometers after kilometers, and thus access to new areas, the same is not valid for most toads. In this case, we recommend that the projections be viewed with caution and not as empirical evidence in treating the future of these animals because of the possible overestimated probabilities in P_M_(g) in the BAM diagram [40].

Given the exponentially growth of the human population and its increasing demands for resources, environmental preservation/conservation areas have recently been understood and designed as a safeguard of wildlife areas and nature strongholds for future generations [67]. There are many reasons that led to the development of the concept of legally protected areas, as well as the justifications to convince governments of the importance of preservation/conservation areas [68, 69]. No doubt, environmental preservation/conservation areas must ensure the protection of species and ecosystems, so that they can still exist in the medium and long term [70]. However, will such areas be effective in protecting these species?

In the particular case of ceratophrids, according to our results, by the year 2080, most of the distribution area of 50% of these frog species will be still included in environmental preservation/conservation areas, which means that half of them will not be vulnerable in the next 65 years. However, this situation becomes alarming when considering habitat destruction coupled with the lack of legal support of protected areas, and in this case, it is very possible that four species will be almost extinct by the turn of this century: *C*. *aurita*, *C*. *ornata* and *C*. *stolzmanni* (see data of the IUCN). If there is a possibility of this happening to ceratophrids, animals that are well adapted to water stress (because of climate changes over the past 140 000 years), the chances of other species of the global amphibiofauna of becoming extinct or extremely vulnerable are much higher. Recent studies showed that the world herpetofauna will endure large losses by 2080 due to global climate change, disease and defaunation caused by humans [65, 71–74].

Much of the optimism of our future projections for ceratophrids are, at best, unlikely scenarios, especially when considering the rapid pace of degradation of habitats and the lack of effectiveness of environmental preservation/conservation areas in the long term [75]. This leads us to conclude that although we may consider a likely limit to the future expansion of distribution for these amphibians, our results suggest a broad expansion of dry climates in South America in the XXII century and hence retraction of forested environments, similar to what occurred during the Pleistocene [56, 57, 76], differing only by a larger decline in diversity, and not only of ceratophrids, around the turn of the century [65].

Other studies have also indicated losses in diversity, especially when it remains confined to specific areas, mainly due to effects of habitat fragmentation and climate change [75, 77–79]. Thus, the effectiveness and functionality of long-term environmental preservation/conservation areas is something very questionable, and that was also what we found out from our results. For example, only in relation to *C*. *aurita*, environmental preservation/conservation areas currently comprise 48.24% of the total distribution of this species, and official data indicate that the Atlantic Forest is under an accelerated rate of destruction [80], including within its own ecological reserves, because of illegal deforestation [81, 82]. This suggests the possibility that by the second half of the twenty-first century all endemic species of the Atlantic Forest (and those that there are distributed) are in serious risk [79, 83, 84], including *C*. *aurita*. With this in mind, one of the challenges will be to know with relative certainty, what would be the appropriate minimum distribution size to provide viability, in this case Ceratophryidae populations, and ensure the future of their species. This question can only be answered by studies in population dynamics.

When developing public policies for the demarcation of environmental reserve areas, the last thing that is contemplated is if the geographical space is appropriate or not for the viability of populations of species, whether animal or plant. This is a subject that does not arouse much interest or popular mobilization, so the areas are generally designed with regard to tourism potential and fitted to private property areas and/or land of economic interest [85]. Moreover, with a serious aggravating future, environmental reserves may give way with changes in economic interests and also increased demands for resources. This will aggravate even further the problem of the conservation of species and generate a serious impasse; therefore, to be viable, the environmental preservation/conservation areas will need to be adjusted as the spatial distribution of biodiversity changes in the near future [86]. This will be necessary if we want these reserves to be functional by offering guarantees of future existence of the species they harbor and consequently also for future generations of humans. But how will this be possible with the massive and continuous destruction of habitats?

For a long time, both philosophers and eminent scientists have been concerned about climate change, many of them even claiming that there seems to be an optimistic future for biodiversity as a whole, including ourselves [87, 88]. Furthermore, the current political and economic reality imposes difficult barriers to overcome [89, 90]. To meet the growing human and livestock demands, it has been necessary to destroy wilderness areas and expand or create new spaces for agriculture in recent centuries. If this scenario continues, and it looks that way, any measure that promises to ensure spatial plasticity to environmental protection areas (albeit restricted) will be more like hope.

Preventive measures need to be urgently developed and implemented so that they can minimize this impasse. However, this is not possible without a radical measure, for example, changing the way we see environmental preservation/conservation areas: from inert objects to dynamic ones, change on the current politico-economic model, making the people aware of the use of natural resources, and restructuring of the urban mechanism from dysfunctional to sustainable [91–93].

The survival of the human species will require a new social model that is based on cooperativity, the responsible and equitable distribution of resources, as well as an education based on the real needs of human society and other species. This will force us to think differently, to reshape our interpersonals relations and to see nature, not as a thing which we preyed upon for centuries, but as a part of ourselves. Only then can we ensure a future not only for ceratophrids but also for all other species and ecosystems.

## Supporting information

S1 File Extended description of the methods. (DOCX)

S1 Fig Retraction dynamics and expansion of the distribution area of *Ceratophrys aurita* according to the niche modeling method. Last interglacial (A); Last glacial maximum (B) and Current (C). (A): 12,163 km2; (B): 34,958 km2 and (C): 11,301 km2. Training data: AUC = 0.985 (A); AUC = 0.986 (B) and AUC = 0.988 (C). Test data: AUC = 0.985 (A); AUC = 0.973 (B) and AUC = 0.986.
(TIF)

S2 Fig Retraction dynamics and expansion of the distribution area of *Ceratophrys calcarata* according to the niche modeling method. Last interglacial (A); Last glacial maximum (B) and Current (C). (A): 7,287 km2; (B): 27,623 km2 and (C): 7,298 km2. Training data: AUC = 0.996 (A); AUC = 0.991 (B) and AUC = 0.997 (C). Test data: AUC = 1:00 (A); AUC = 0.710 (B) and AUC = 0.997.
(TIF)

S3 Fig Retraction dynamics and expansion of the distribution area of *Ceratophrys cornuta*, according to the niche modeling method. Last interglacial (A); Last glacial maximum (B) and Current (C). (A): 59,979 km^2^; (B): 166,127 km^2^ and (C): 76,329 km^2^. Training data: AUC = 0.962 (A); AUC = 0.936 (B) and AUC = 0.956 (C). Test data: AUC = 0.892 (A); AUC = 0.919 (B) and AUC = 0.929.
(TIF)

S4 Fig Retraction dynamics and expansion of the distribution area of *Ceratophrys cranwelli*, according to the niche modeling method. Last interglacial (A); Last glacial maximum (B) and Current (C). (A): 67,880 km^2^; (B): 82,321 km^2^ and (C): 69,362 km^2^. Training data: AUC = 0.966 (A); AUC = 0.950 (B) and AUC = 0.956 (C). Test data: AUC = 0.930 (A); AUC = 0.942 (B) and AUC = 0.942 (C).
(TIF)

S5 Fig Retraction dynamics and expansion of the distribution area of *Ceratophrys joazeirensis*, according to the niche modeling method. Last interglacial (A); Last glacial maximum (B) and Current (C). (A): 14,574 km^2^; (B): 62,883 km^2^ and (C): 33,162 km^2^. Distribution areas shown in the most western parts of the continent are unlikely and represent analysis artifacts resulting from Grinnellian niche concept. Training data: AUC = 0.989 (A); AUC = 0.968 (B) and AUC = 0.984 (C). Test data: AUC = 0.995 (A); AUC = 0.976 (B) and AUC = 0.984 (C).
(TIF)

S6 Fig Retraction dynamics and expansion of the distribution area of *Ceratophrys ornata*, according to the niche modeling method. Last interglacial (A); Last glacial maximum (B) and Current (C). (A): 22,657 km^2^; (B): 54,701 km^2^ and (C): 25,563 km^2^. Distribution areas indicated in the extreme south of the continent may be due to analysis artifacts resulting from Grinnellian niche concept. Training data: AUC = 0.994 (A); AUC = 0.982 (B) and AUC = 0.991 (C). Test data: AUC = 0.991 (A); AUC = 0.988 (B) and AUC = 0.984 (C).
(TIF)

S7 Fig Retraction dynamics and expansion of the species' distribution area of *Ceratophrys stolzmanni*, according to the niche modeling method. Last interglacial (A); Last glacial maximum (B) and Current (C). (A): 746 km^2^; (B): 4,729 km^2^ and (C): 595 km^2^. Distribution areas indicated in the northern part of the continent are unlikely and represent analysis artifacts resulting from Grinnellian niche concept. Training data: AUC = 1.000 (A); AUC = 0.999 (B) and AUC = 1.000 (C). Test data: AUC = 1.000 (A); AUC = 0.987 (B) and AUC = 0.996 (C).
(TIF)

S8 Fig Retraction dynamics and expansion of the species' distribution area *Chacophrys pierottii*, according to the niche modeling method. Last interglacial (A); Last glacial maximum (B) and Current (C). (A): 68,824 km^2^; (B): 43,109 km^2^ and (C): 58,920 km^2^. Distribution areas indicated in the extreme west of the continent are unlikely and represent analysis artifacts resulting from the Grinnellian niche concept. Training data: AUC = 0.989 (A); AUC = 0.991 (B) and AUC = 0.971 (C). Test data: AUC = 0.940 (A); AUC = 0.917 (B) and AUC = 0.995.
(TIF)

S9 Fig Retraction dynamics and expansion of the distribution area of *Lepidobatrachus laevis*, according to the niche modeling method. Last interglacial (A); Last glacial maximum (B) and Current (C). (A): 37,905 km^2^; (B): 31,409 km^2^ and (C): 31,822 km^2^. Training data: AUC = 0.992 (A); AUC = 0.991 (B) and AUC = 0.994 (C). Test data: AUC = 0.982 (A); AUC = 0.994 (B) and AUC = 0.940 (C).
(TIF)

S10 Fig Retraction dynamics and expansion of the range of *Lepidobatrachus asper*, according to the niche modeling method. Last interglacial (A); Last glacial maximum (B) and Current (C). (A): 59,958 km^2^; (B): 74,262 km^2^ and (C): 55 569 km^2^. Training data: AUC = 0.979 (A); AUC = 0.980 (B) and AUC = 0.979 (C). Test data: AUC = 0.962 (A); AUC = 0.970 (B) and AUC = 0.954 (C).
(TIF)

S11 Fig Retraction dynamics and expansion of the distribution area of *Lepidobatrachus llanensis*, according to niche modeling method. Last interglacial (A); Last glacial maximum (B) and Current (C). (A): 31,170 km^2^; (B): 29 288 km^2^ and (C): 24,105 km^2^. Distribution areas indicated in the western part of the continent are unlikely and represent analysis artifacts resulting from the Grinnellian niche concept. Training data: AUC = 0.994 (A); AUC = 0.989 (B) and AUC = 0.996 (C). Test data: AUC = 0.985 (A); AUC = 0.996 (B) and AUC = 1.000 (C).
(TIF)

S12 Fig AUC curves and test Jackknife of the environmental variables for all of the climate models of *Lepidobatrachus asper*. (TIF)

S13 Fig AUC curves and test Jackknife of the environmental variables for all of the climate models of *Ceratophrys aurita*. (TIF)

S14 Fig AUC curves and test Jackknife of the environmental variables for all of the climate models of *Ceratophrys calcarata*. (TIF)

S15 Fig AUC curves and test Jackknife of the environmental variables for all of the climate models of *Chacophrys pierottii*. (TIF)

S16 Fig AUC curves and test Jackknife of the environmental variables for all of the climate models of *Ceratophrys cornuta*. (TIF)

S17 Fig AUC curves and test Jackknife of the environmental variables for all of the climate models of *Ceratophrys cranwelli*. (TIF)

S18 Fig AUC curves and test Jackknife of the environmental variables for all of the climate models of *Ceratophrys joazeirensis*. (TIF)

S19 Fig AUC curves and test Jackknife of the environmental variables for all of the climate models of *Lepidobatrachus laevis*. (TIF)

S20 Fig AUC curves and test Jackknife of the environmental variables for all of the climate models of *Lepidobatrachus llanenis*. (TIF)

S21 Fig AUC curves and test Jackknife of the environmental variables for all of the climate models of *Ceratophrys ornata*. (TIF)

S22 Fig AUC curves and test Jackknife of the environmental variables for all of the climate models of *Ceratophrys stolzmannii*. (TIF)

S1 Table Distribution data of the species of Ceratophyidae obtained of literature procedence. (XLSX)

S2 Table Distribution data of the species of Ceratophyidae obtained of museum procedence. (XLSX)
