# Supplementary material for: Effect of climate change on distribution of species of common horned frogs in South America
Source: PLoS One. 2018 Sep 12;13(9):e0202813. doi: 10.1371/journal.pone.0202813 (PMC6135375; doi:10.1371/journal.pone.0202813)
Supplement: S1 File — (DOCX) [file pone.0202813.s001.docx]

**Supporting Information** for

# Effect of Climate Change on Distribution of Species of Common Horned Frogs in South America

Kleber da Silva Vieira^1,2¶^*; Paulo Fernandes Guedes Montenegro^1&^; Gindomar Gomes Santana^3&^ Washington Luiz da Silva Vieira^1¶^

^1^Laboratório de Ecofisiologia Animal do Departamento de Sistemática e Ecologia da Universidade Federal da Paraíba, João Pessoa, Paraíba; Brasil.

^2^**Bolsista CNPq pelo Programa de Desenvolvimento Científico e Tecnológico Regional (FAPESQPB)/Universidade Estadual da Paraíba, Campina Grande,** Paraíba; **Brasil.**

^3^**Bolsista do Programa Nacional de Pós-Doutorado (PNPD), Programa de Pós-Graduação em Ecologia e Conservação (PPGEC)/CCBS, Universidade Estadual da Paraíba, Campina Grande,** Paraíba;  **Brasil.**

***Corresponding autor:**

E-mail: [ksvieira04@yahoo.com.br](mailto:ksvieira04@yahoo.com.br)

^¶^ These authors contributed equally to this work.

^&^These authors also contributed equally to this work.

# Extended description of the Methods

The Data of the Distribution

All information on the distribution of the species studied was obtained from specimens preserved in scientific collections (presence data). The data came from, in this case, the following sources: information of specimens examined contained on labels and/or records of museum collections, as well as scientific articles (S1 and S2 Tables). The main problem here was to recognize the likely misidentifications. Thus, to reduce the probability of type I error (considering specimen X as being of the species X, when in fact it is not) and type II error (considering specimen X as not being of the species X, when in reality it is), the following procedures were taken:

a) Animals examined and analyzed on the spot had their characteristics compared to original descriptions of the species attributed to them - morphological criteria of species ([1](#_ENREF_1)). When this was not possible, the diagnoses replaced descriptions. The two sources of data were often used;

b) The current occupation area of the specimens analyzed served as a parameter for those referred to voucher books and scientific articles (specimens not examined in situ). When areas coincided with the known distribution, the premise was accepted that a certain specimen belonged to a particular species. This agreement was based not only on overlapping biogeographic information; the Buffon principle was also assumed: each species possesses characteristics that are in accordance with the type of environment in which it is located, and that such characteristics are due to a synergistic effect between character and environment ([2](#_ENREF_2)). That is, if *C. joazeirensis* is a typical species of the Caatinga ([3](#_ENREF_3)), we assume that it does not occur in the Amazon Rainforest. If there is any record of *C. joazeirensis* in the latter, to be safe, we reject such identification and treat this record as being probably *C. cornuta* (only species widely distributed in the Amazon).

For specimens that did not have precise site or geographic coordinates in their records, that information was obtained from the city and/or district near the collection site, up to 10 km. This was make to accommodate spatial inaccuracies in the species occurrences, without causing profound distortions on the final data of geographic distribution, since the species studied here are of restricted vagility. Such information was found through the GEOlocate web application ([4](#_ENREF_4)).

The maps of potential distribution

The maps of potential distribution of past, present and future of species of the family Ceratophryidae (S1-S11 Figs) were generated through the DIVA-GIS software ([5](#_ENREF_5)) and MaxEnt ([6](#_ENREF_6)), performing it run for each species separately. The MaxEnt use both pseudoabsence and presence data randomly sampled from the calibration area and the Bioclim is an envelope-mode method that depict sites that are located within the geografical space potentially occupied by a species. These geographic information systems (GIS) are able to model niches on the basis of environmental data of the places where a particular species has been found - points of presence ([7](#_ENREF_7), [8](#_ENREF_8)). The MaxEnt examines certain environmental conditions linking them to real presence points and random points of a sub-sample, selecting an area of geographic space where the species potentially occurs ([9](#_ENREF_9)). The ratio of the species to the environment in DIVA-GIS is made using the BIOCLIM algorithm, which employs a simple non-deterministic and transparent approach that is suitable for presence data in large sets of environmental information ([10](#_ENREF_10), [11](#_ENREF_11)).

We chose to use the DIVA-GIS and MaxEnt software because they are at the same time simpler and precise for the particularities the species studied here (restricted dispersal properties and niche known, although limited available data), without sacrificing your graphical output nor consistence of the predictions, especially MaxEnt ([12](#_ENREF_12), [13](#_ENREF_13)), compared to other available software when in specific circumstances ([14](#_ENREF_14)), principally for species with low vagility and dispersion capacity restricted, as amphibians are when compared to others vertebrates species such several types birds and mammals.

In our study the bioclimatic data of the past, present and future are continuous variables that are part of GIS layers obtained from the WorldClim portal ([15](#_ENREF_15)). They were derived from the monthly temperature and rainfall values in order to generate more biologically meaningful variables. In our study, the bioclimatic variables represent annual trends (e.g., mean annual temperature, annual precipitation) seasonality (e.g., annual range in temperature and precipitation) and extreme or limiting environmental factors (e.g., temperature of the coldest and warmest month, and precipitation of the wet and dry quarters).

The layers representing current climates are in a resolution of 2.5 arc-minutes (~4.5 km^2^ at the equator), the which contained bioclimatic data from the 1950s to the present. To predict the distribution of species of Ceratophryidae in future climates, we used climate models with a resolution of 30 arc-seconds (0.93 x 0.93 = 0.86 km^2^ at the equator) for 2050 and 2080 of the Canadian Centre for Climate Modelling and Analysis – CCCMA (CGCM4/CMIP5). Emission scenarios used were A2 and B2 ([16](#_ENREF_16), [17](#_ENREF_17)). This was done to predict the distributions in reasonably optimistic (B2) and/or pessimistic (A2) scenarios of global climate change according to the Intergovernmental Panel on Climate Change - IPCC ([18](#_ENREF_18), [19](#_ENREF_19)).

The distribution study of the species of Ceratophryidae in paleoclimatic conditions was conducted in the GIS layers of the Last interglacial (~ 120 000-140 000 BP) and the Last glacial maximum (~ 21 000 years BP). The first layer is in a resolution of 30 arc-seconds ([20](#_ENREF_20)), the other at 2.5 arc-minutes. The latter was generated by the Paleoclimate Modelling Intercomparison Project Phase II (PMIP2). The original data was made available by CMIP5, being downscaled and calibrated (bias corrected) using WorldClim 1.4 as baseline 'current' climate. We performed this analysis in attempt to verify how the area of habitat suitability of ceratophrids probably changed (shifted, widened or decreased) over time. This information is of great importance for large-scale conservation plans, since it is predictive of key regions where species are likely to suffer the effects of habitat destruction ([21](#_ENREF_21)).

MaxEnt Calibration

We chose to change the automatic options of MaxEnt for better software performance when testing the bioclimatic variables ([22](#_ENREF_22)). At this point of the study, randomizations were produced by random seed, where we sought to test the original information of the samples, allowing enough time to achieve convergence of the variables, to avoid committing errors of commission or omission by generating potential distributions in the absence of an independent set of data. Thus, we used a maximum of 5000 repetitions and 25 randomizations in the percentage test (avoiding the interference of the training data) to evaluate the performance of the models, as well as 15 replicates to calculate the mean of the results during this process. The sampling technique used in our analysis was subsampling (replicate run type). We did not adjust the sampling radius, and the convergence threshold was kept at 0.00001. The presence data duplicates (multiple records for the same species in the same grid cell), were removed by default setting.

To discriminate the individual percentage contribution of the model (regularized training gain), we applied the Jackknife test to the model in all environmental layers and also generated predictive contribution tables for the variables (S12-S22 Figs). These tables were constructed because the particular predictive power was easily checked for each distribution variable of the species studied ([22](#_ENREF_22)).

The performance of each estimated model was generated by the "area under curve" (AUC). In this case, AUC values equal or close to 1 indicate excellent accuracy. Values equal to or less than 0.5 are predictive results that are not as good as those obtained randomly ([23](#_ENREF_23)). AUC is a probability estimator generally used as a gauge in tests that predict the accuracy of a model ([24](#_ENREF_24)). The use of AUC as a standard precision tool should be taken with caution, given that the degree of accuracy of this estimator is sometimes reported as being dependent on the sample size, where the smaller the sample used, the lower the accuracy ([25](#_ENREF_25)). The application of ROC AUC is still controversial ([26](#_ENREF_26)), but some studies suggest that it is essentially a very reliable measure of accuracy when compared to other estimators ([27](#_ENREF_27), [28](#_ENREF_28)).

Because of Diva-GIS software sacrifices the accuracy and realism of the predicted distribution according to the generality of the model produced ([29](#_ENREF_29)), we chose not to show the results with BIOCLIM, restricting us to use only the information gathered to discuss the form of relationship between the species studied and environmental conditions of the area that it occupies. On the other hand, the assessment of changes in the distribution ceratophrid species was facilitated by overlapping and subtraction of polygons and raster layers made in Diva-GIS.

To avoid ambiguity and a wide range of settings ([30](#_ENREF_30)), we decided to apply the term areapause to spatial limits (outlines) of the suitable area of the species. The proposal of this term, inspired in the astronomical term heliopause, suggests that the distribution is stopped because of some ecological pressure and because the species is no longer able to adjust to certain areas of the geographical space. Areapause is a virtual term to most outer border of the distribution (varying according of method and descriptors). Different factors can determine the spatial limit of the distribution of a species. In our study, it was shown by potential distribution, indicted by abiotic factors, here designed through of climatic variables - Grinnellian niche ([31](#_ENREF_31)).

The rasters containing the models of the potential distribution areas of ceratophrids have also been overlapping to polygons of geographical areas which delimited environmental preservation/conservation areas in South America ([32](#_ENREF_32)). This was done to determine how the effective distribution of these frogs was or wasn’t inside in areas protected by law, facilitating in this way, future categorization of the conservation status ([33](#_ENREF_33)). Thus, the size of areas (in km^2^) was estimated from the pixels of the coverage maps generated in raster format (DIVA-GIS). In this case, it is likely that some values were overestimated, comprising areas where the species are known not to occur (due to geographical barriers), commission errors, or varied according emission scenario.

Bioclimatic Variables according the Global Climate Data ([15](#_ENREF_15))

The bioclimatic variables were derived from the monthly temperature and rainfall values. The variables used here are coded as follows:

BIO1 = Annual Mean Temperature
BIO2 = Mean Diurnal Range (Mean of monthly (max temp - min temp))
BIO3 = Isothermality (BIO2/BIO7) (* 100)
BIO4 = Temperature Seasonality (standard deviation *100)
BIO5 = Max Temperature of Warmest Month
BIO6 = Min Temperature of Coldest Month
BIO7 = Temperature Annual Range (BIO5-BIO6)
BIO8 = Mean Temperature of Wettest Quarter
BIO9 = Mean Temperature of Driest Quarter
BIO10 = Mean Temperature of Warmest Quarter
BIO11 = Mean Temperature of Coldest Quarter
BIO12 = Annual Precipitation
BIO13 = Precipitation of Wettest Month
BIO14 = Precipitation of Driest Month
BIO15 = Precipitation Seasonality (Coefficient of Variation)
BIO16 = Precipitation of Wettest Quarter
BIO17 = Precipitation of Driest Quarter
BIO18 = Precipitation of Warmest Quarter
BIO19 = Precipitation of Coldest Quarter

This scheme follows that of ANUCLIM, except that for temperature seasonality the standard deviation was used because a coefficient of variation does not make sense with temperatures between -1 and 1).

# References

1. de Queiroz K. The General Lineage Concept of Species, Species Criteria, and the Process of Speciation. In: Howard DJ, Berlocher SH, editors. Endless Forms: Species and Speciation. Oxford: Oxford Universioty Press; 1998. p. 57-75.

2. Cox CB, Moore PD. Biogeography: An Ecological and Evolutionary Approach. 7^a^ ed. Oxford: Blackwell Publishing; 2005.

3. Mercadal IT. *Ceratophrys joazeirensis* sp. n. (Ceratophryidae, Anura) del noreste de Brasil. Amphibia-Reptilia. 1986;7:313-34.

4. Rios NE, Bart Jr HL, Abibou D. GEOLocate Web Application - A Platform for Georeferencing Natural History Collections Data Belle Chasse, LA Tulane University Biodiversity Research Institute; 2013 [cited 2013 10/09]. Available from: <http://www.museum.tulane.edu/geolocate/default.html>.

5. Hijmans RJ, Guarino L, Jarvis A, O'Brien R, Mathur P, Bussink C, et al. DIVA-Gis Version 7.5. Bioversity International, the International Potato Center, the International Rice Research Institute, the University of California-Berkeley Museum of Vertebrate Zoology, and others; 2005. p. 11p.

6. Phillips S, Dudik M, Schapire R. Maximum Entropy Modeling of Species Geographic Distributions. MaxEnt Version 3.3.3e. 3.3.3e ed. Princeton: AT&T Labs-Research, Princeton University, and the Center for Biodiversity and Conservation, American Museum of Natural History; 2006.

7. Phillip SJ, Anderson RP, Schapire RE. Maximum Entropy Modeling of Species Geographic Distributions. Ecological Modelling. 2006;190:231-59.

8. Scheldeman X, van Zonneveld M. Training Manual on Spatial Analysis of Plant Diversity and Distribution. Rome - Italy: Bioversity International 2010. 179 p.

9. Phillip SJ, Dudík M. Modeling of Species Distributions with Maxent: new extensions and a comprehensive evaluation. Ecography. 2007;31:161-75.

10. Guisan A, Zimmermann NE. Predictive Habitat Distribution Models in Ecology. Ecological Modelling. 2000;135:147-86.

11. Fernández M, Cole D, Heyer WR, Reichle S, O de Sá  R. Predicting *Leptodactylus* (Amphibia, Anura, Leptodactylidae) distributions: broad-ranging versus pathily distributed species using a presence-omly environmental niche modeling technique. South American Journal of Herpetology. 2009;4(2):103-16.

12. Hernandez PA, Graham CH, Master LL, Albert DL. The Effect of Sample Size and Species Characteristics on Performance of Different Species Distribution Modeling Methods. Ecography. 2006;29:773-85.

13. Giovanelli JGR, de Siqueira MF, Haddad CFB, Alexandrino J. Modeling a Spatially Restricted Distribution in the Neotropics: How the Size of Calibration Area Affects the Performance of Five Presence-only Methods. Ecological Modelling. 2010;221:215-24.

14. Qiao H, Soberón J, Peterson AT. No Silver Bullets in Correlative Ecological Niche Modelling: insights from testing among many potential algorithms for niche estimation. Methods in Ecology and Evolution. 2015;6(10):1126-36.

15. Hijmans RJ, Cameron S, Parra J, Jones P, Jarvis A, Richardson K. WorldClim - Global Climate Data Berkeley: Museum of Vertebrate Zoology, University of California; 2005 [cited 2013 september, 26]. Available from: <http://www.worldclim.org/about>.

16. Nakičenovič N, Davidson G, Grübler A, Kram T, La Rovere EL, Metz B, et al. A Special Report of IPCC Working Group III - Emissions Scenarios: Intergovernmental Panel on Climate Change; 2000.

17. Govindasamy B, Duffy PB, Coquard J. High-Resolution Simulations of Global Climate, part 2: effects of increased greenhouse cases. Climate Dynamics. 2003;21:391-404.

18. Watson RT, Albritton DL, Barker T, Bashmakov IA, Canziani O, Christ R, et al. Climate Change 2001: Synthesis Report. 2001 24-29 September 2001. Report No.: VIII.

19. Alley RB, Berntsen T, Bindoff NL, Chen Z, Chidthaisong A, Friedlingstein P, et al. A Report of Working Group I of the Intergovernmental Panel on Climate Change - Summary for Policymakers. In: Qin D, Manning M, Chen Z, Marquis M, Averyt KB, Tignor M, et al., editors. Climate Change 2007: The Physical Science Basis. Cambridge, United Kingdom and New York, NY, USA: Cambridge University Press; 2007. p. 18.

20. Otto-Bliesner B, Marshall SJ, Overpeck JT, Miller GH, Hu A. Simulating Arctic Climate Warmth and Icefield Retreat in the Last Interglaciation. Science. 2006;311:1751.

21. Tôrres NM, Vercillo UE. How Can Species Distribution Modeling Tools Support Government Actions? Natureza & Conservação. 2012;10(2):228-30.

22. Young N, Carter L, Evangelista P. A MaxEnt Model v3.3.3e Tutorial (ArcGis v10). Colorado: Natural Resource Ecology/National Institute of Invasive Species/Colorado State University; 2011. p. 1-30.

23. Fielding AH, Bell JF. A Review of Methods for the Assessment of Prediction Errors in Conservation Presence/absence Models. Environmental Conservation. 1997;24(1):38-49.

24. Pearce J, Ferrier S. Evaluating the Predictive Performance of Habitat Models Developed Using Logistic Regression. Ecological Modelling. 2000;133:225-45.

25. Hanczar B, Hua J, Sima C, Weinstein J, Bittner M, Dougherty E. Small-sample Precision of ROC-related Estimates. Bioinformatics. 2010;26(6):822-30.

26. Lobo JM, Jiménez-Valverde A, Real R. AUC: a misleading measure of the performance of predictive distribution models. Global Ecology and Biogeography. 2008;17:145-51.

27. McPherson JM, Jetz W, Rogers DJ. The Effects of Species' Range Sizes on the Accuracy of Distribution Models: ecological phenomenon or statistical artefact? Journal of Applied Ecology. 2004;41:811-23.

28. Peterson AT, Papes M, Soberón J. Rethinking Receiver Operating Characteristic Analysis Applications in Ecological Niche Modeling. Ecological Modelling. 2008;213:63-72.

29. Rangel TF, Loyola RD. Labeling Ecological Niche Models. Natureza & Conservação. 2012;10(2):119-26.

30. Gaston KJ, Fuller RA. The Sizes of Species' Geographic Ranges. Journal of Applied Ecology. 2009;46:1-9.

31. Soberón J. Grinnellian and Eltonian Niches and Geographic Distributions of Species. Ecology Letters. 2007 10:1115-23.

32. IUCN, UNEP. The World Database on Protected Areas (WDPA) Cambridge, UK: UNEP-WCMC; 2014 [cited 2014 05/ may]. Available from: [www.protectedplanet.net](http://www.protectedplanet.net).

33. Paglia AP, de Rezende DT, Koch I, Kortz AR, Donatt C. Species Distribution Models (SDM) in Biodiversity Conservation Strategies and Climate Change Ecosystem Based Adaptation. Natureza & Conservação. 2012;10(2):231-4.
