# Supplementary figures and images for: Effect of climate change on distribution of species of common horned frogs in South America
Source: PLoS One. 2018 Sep 12;13(9):e0202813. doi: 10.1371/journal.pone.0202813 (PMC6135375; doi:10.1371/journal.pone.0202813)

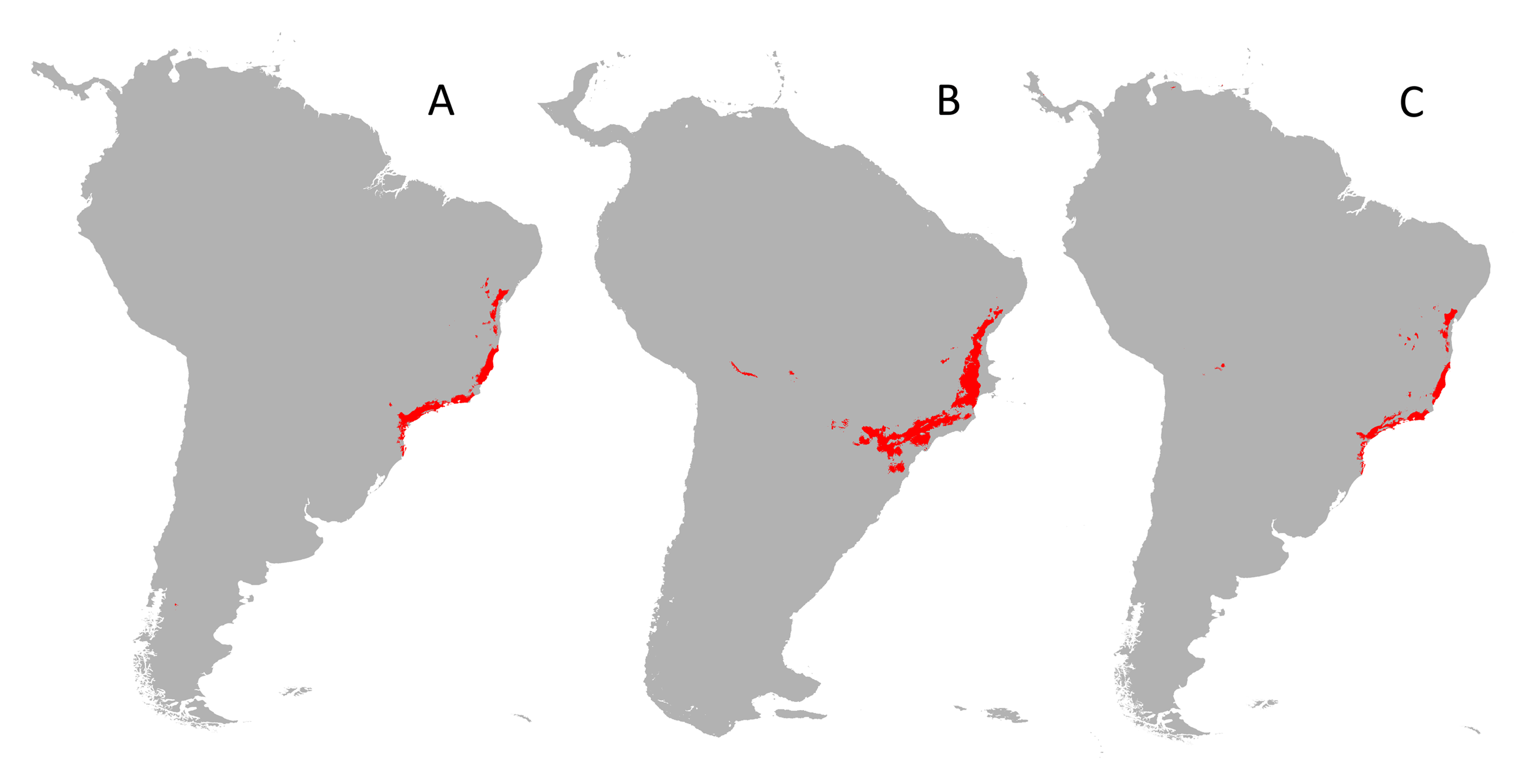

Supplement: S1 Fig — Last interglacial (A); Last glacial maximum (B) and Current (C). (A): 12,163 km2; (B): 34,958 km2 and (C): 11,301 km2. Training data: AUC = 0.985 (A); AUC = 0.986 (B) and AUC = 0.988 (C). Test data: AUC = 0.985 (A); AUC = 0.973 (B) and AUC = 0.986. (TIF) [file pone.0202813.s002.tif]

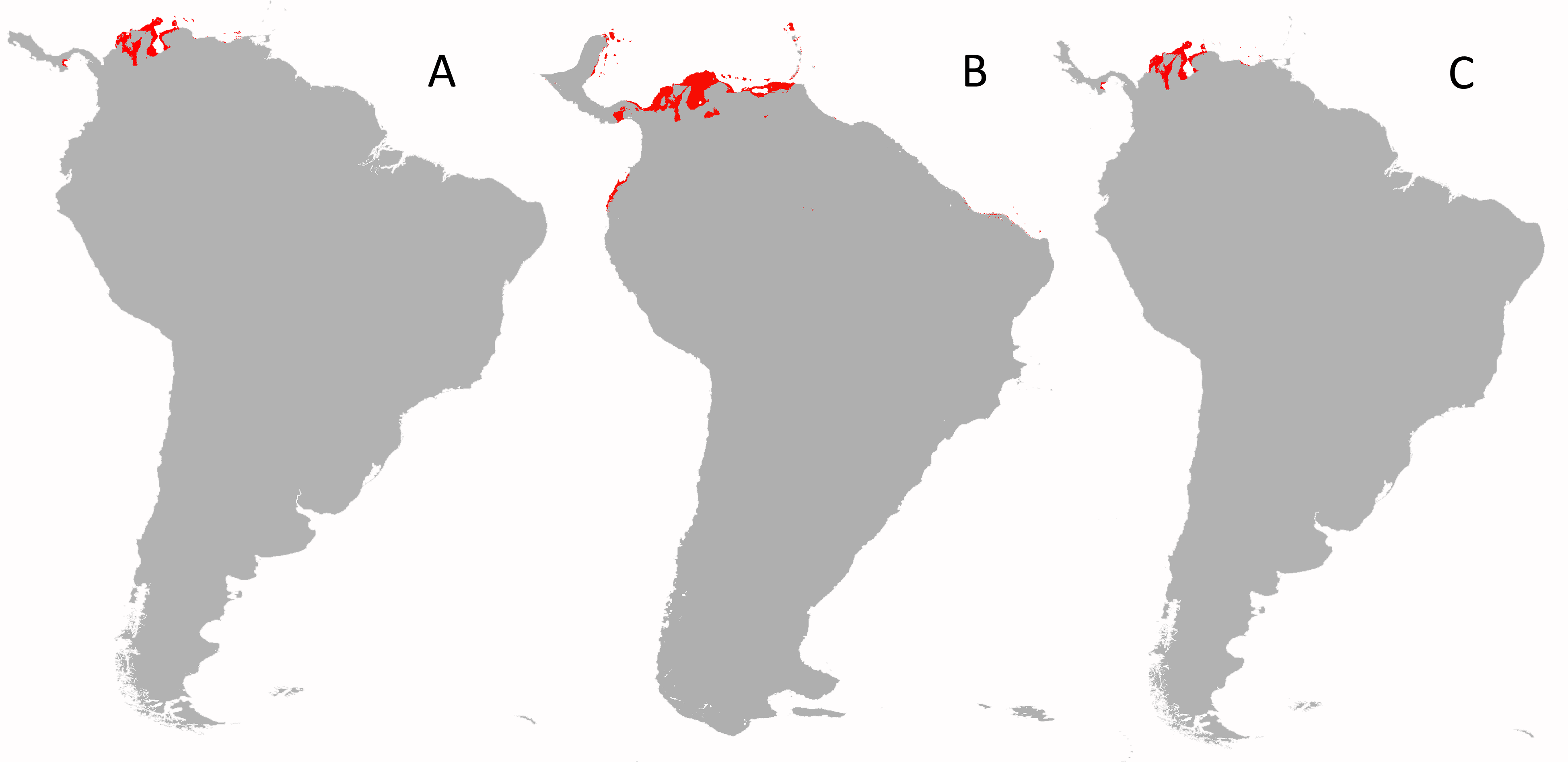

Supplement: S2 Fig — Last interglacial (A); Last glacial maximum (B) and Current (C). (A): 7,287 km2; (B): 27,623 km2 and (C): 7,298 km2. Training data: AUC = 0.996 (A); AUC = 0.991 (B) and AUC = 0.997 (C). Test data: AUC = 1:00 (A); AUC = 0.710 (B) and AUC = 0.997. (TIF) [file pone.0202813.s003.tif]

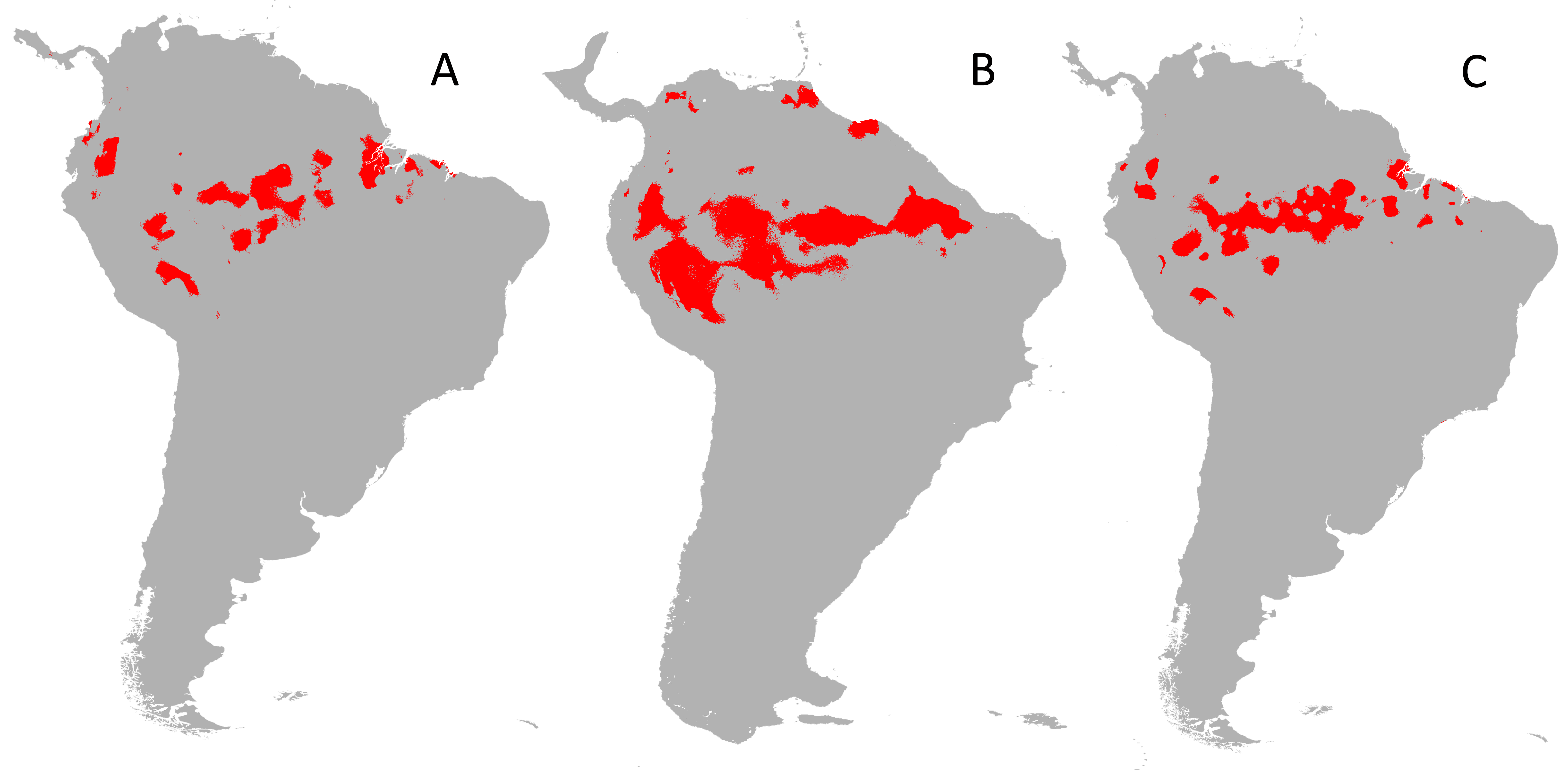

Supplement: S3 Fig — Last interglacial (A); Last glacial maximum (B) and Current (C). (A): 59,979 km2; (B): 166,127 km2 and (C): 76,329 km2. Training data: AUC = 0.962 (A); AUC = 0.936 (B) and AUC = 0.956 (C). Test data: AUC = 0.892 (A); AUC = 0.919 (B) and AUC = 0.929. (TIF) [file pone.0202813.s004.tif]

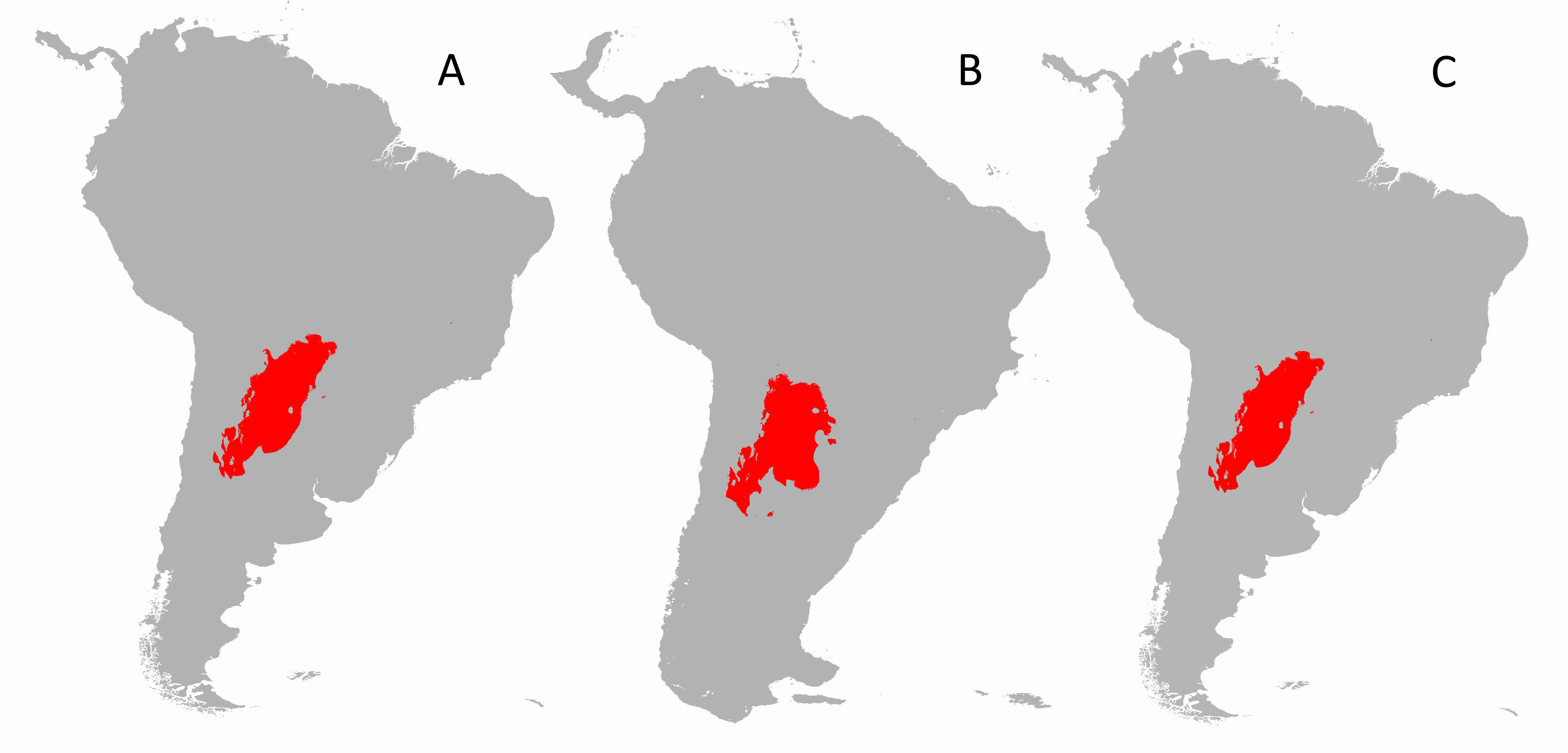

Supplement: S4 Fig — Last interglacial (A); Last glacial maximum (B) and Current (C). (A): 67,880 km2; (B): 82,321 km2 and (C): 69,362 km2. Training data: AUC = 0.966 (A); AUC = 0.950 (B) and AUC = 0.956 (C). Test data: AUC = 0.930 (A); AUC = 0.942 (B) and AUC = 0.942 (C). (TIF) [file pone.0202813.s005.tif]

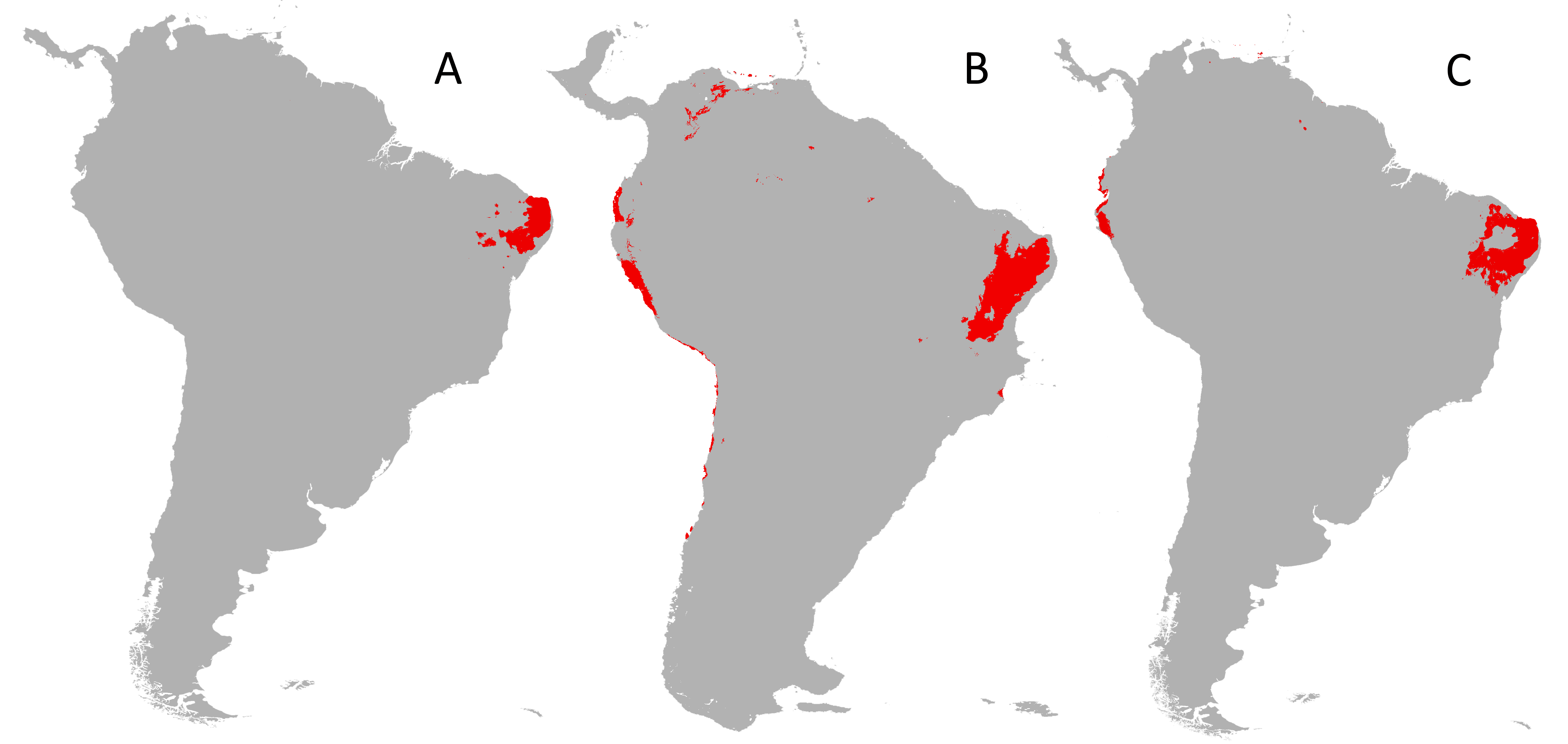

Supplement: S5 Fig — Last interglacial (A); Last glacial maximum (B) and Current (C). (A): 14,574 km2; (B): 62,883 km2 and (C): 33,162 km2. Distribution areas shown in the most western parts of the continent are unlikely and represent analysis artifacts resulting from Grinnellian niche concept. Training data: AUC = 0.989 (A); AUC = 0.968 (B) and AUC = 0.984 (C). Test data: AUC = 0.995 (A); AUC = 0.976 (B) and AUC = 0.984 (C). (TIF) [file pone.0202813.s006.tif]

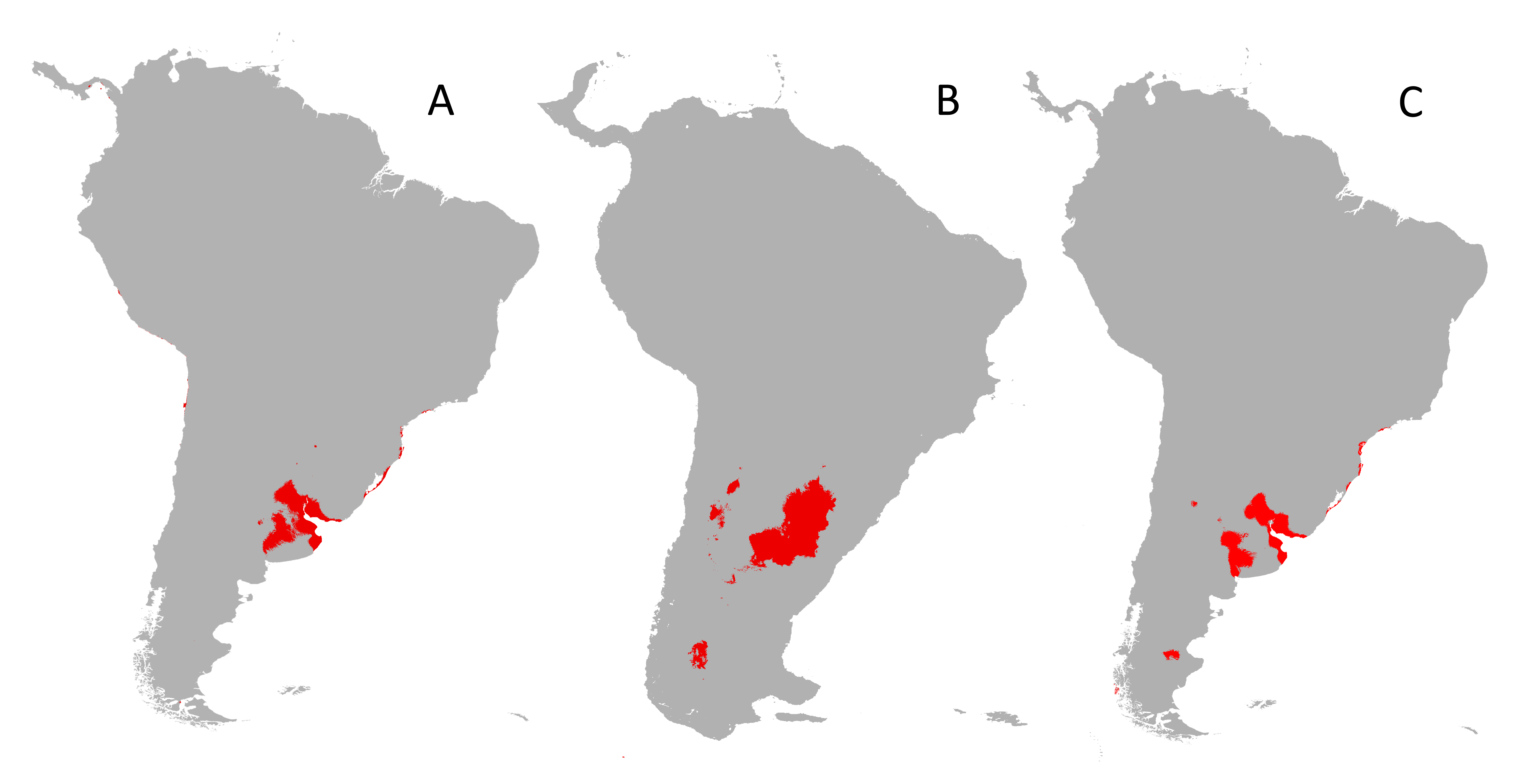

Supplement: S6 Fig — Last interglacial (A); Last glacial maximum (B) and Current (C). (A): 22,657 km2; (B): 54,701 km2 and (C): 25,563 km2. Distribution areas indicated in the extreme south of the continent may be due to analysis artifacts resulting from Grinnellian niche concept. Training data: AUC = 0.994 (A); AUC = 0.982 (B) and AUC = 0.991 (C). Test data: AUC = 0.991 (A); AUC = 0.988 (B) and AUC = 0.984 (C). (TIF) [file pone.0202813.s007.tif]

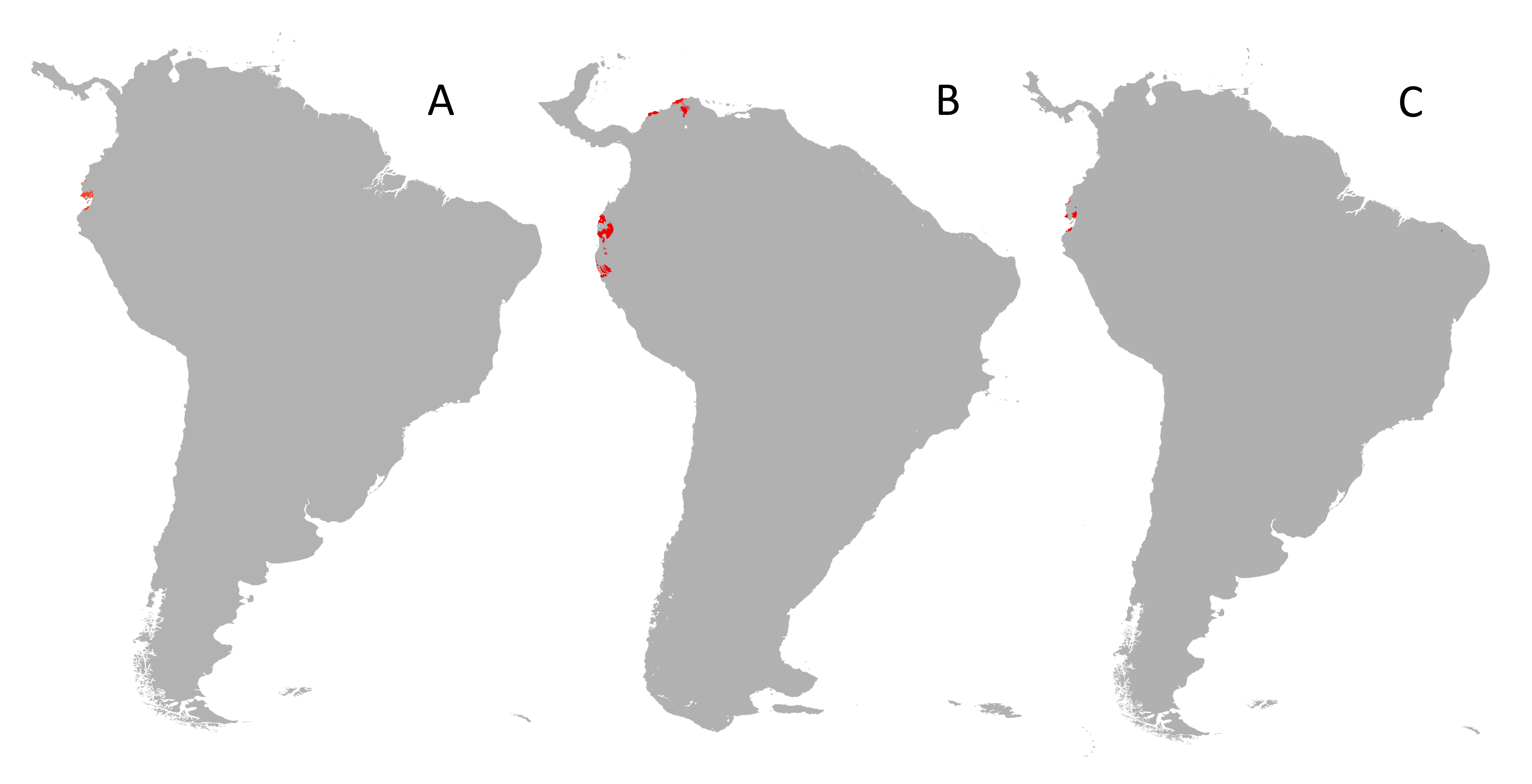

Supplement: S7 Fig — Last interglacial (A); Last glacial maximum (B) and Current (C). (A): 746 km2; (B): 4,729 km2 and (C): 595 km2. Distribution areas indicated in the northern part of the continent are unlikely and represent analysis artifacts resulting from Grinnellian niche concept. Training data: AUC = 1.000 (A); AUC = 0.999 (B) and AUC = 1.000 (C). Test data: AUC = 1.000 (A); AUC = 0.987 (B) and AUC = 0.996 (C). (TIF) [file pone.0202813.s008.tif]

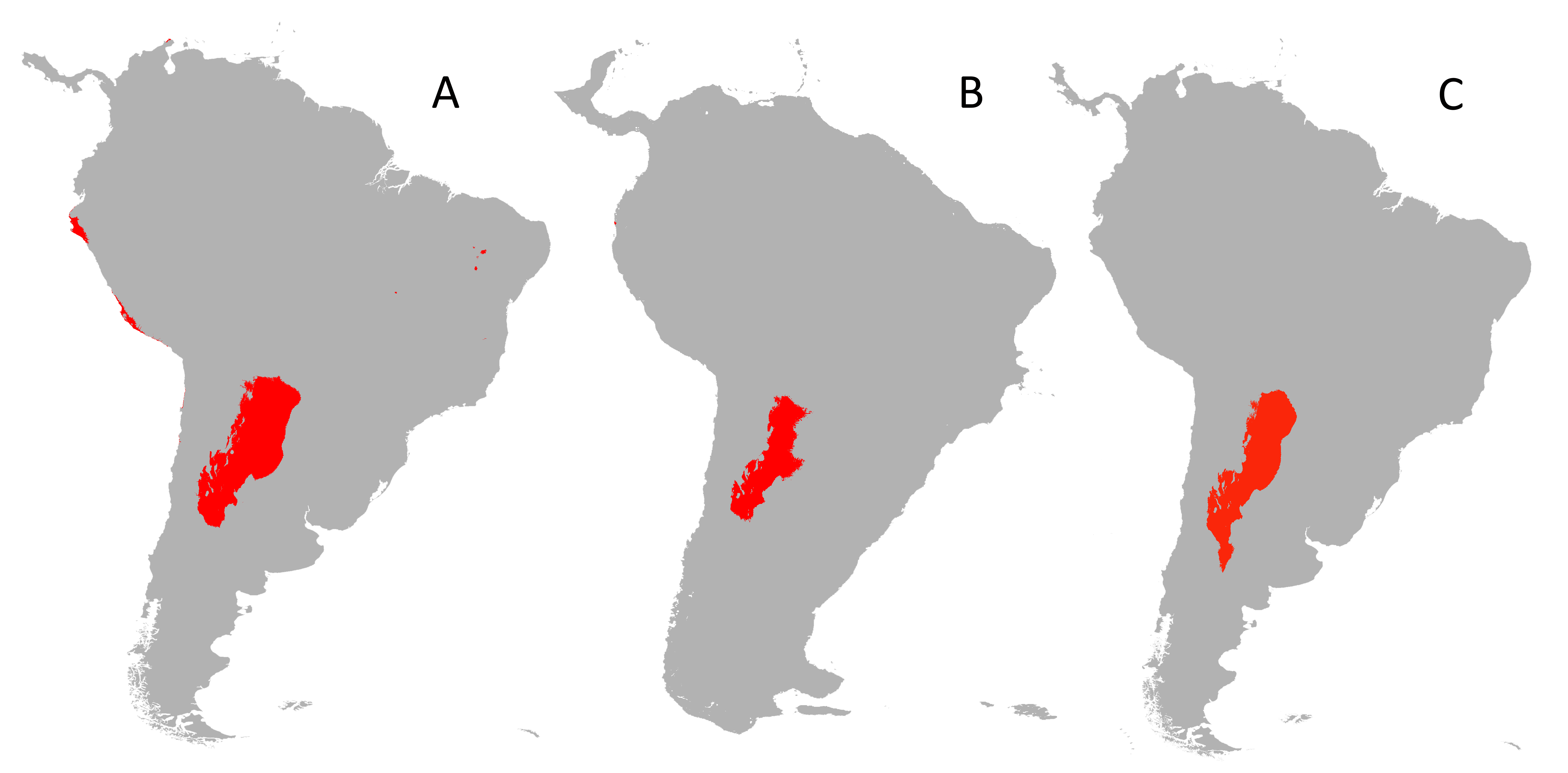

Supplement: S8 Fig — Last interglacial (A); Last glacial maximum (B) and Current (C). (A): 68,824 km2; (B): 43,109 km2 and (C): 58,920 km2. Distribution areas indicated in the extreme west of the continent are unlikely and represent analysis artifacts resulting from the Grinnellian niche concept. Training data: AUC = 0.989 (A); AUC = 0.991 (B) and AUC = 0.971 (C). Test data: AUC = 0.940 (A); AUC = 0.917 (B) and AUC = 0.995. (TIF) [file pone.0202813.s009.tif]

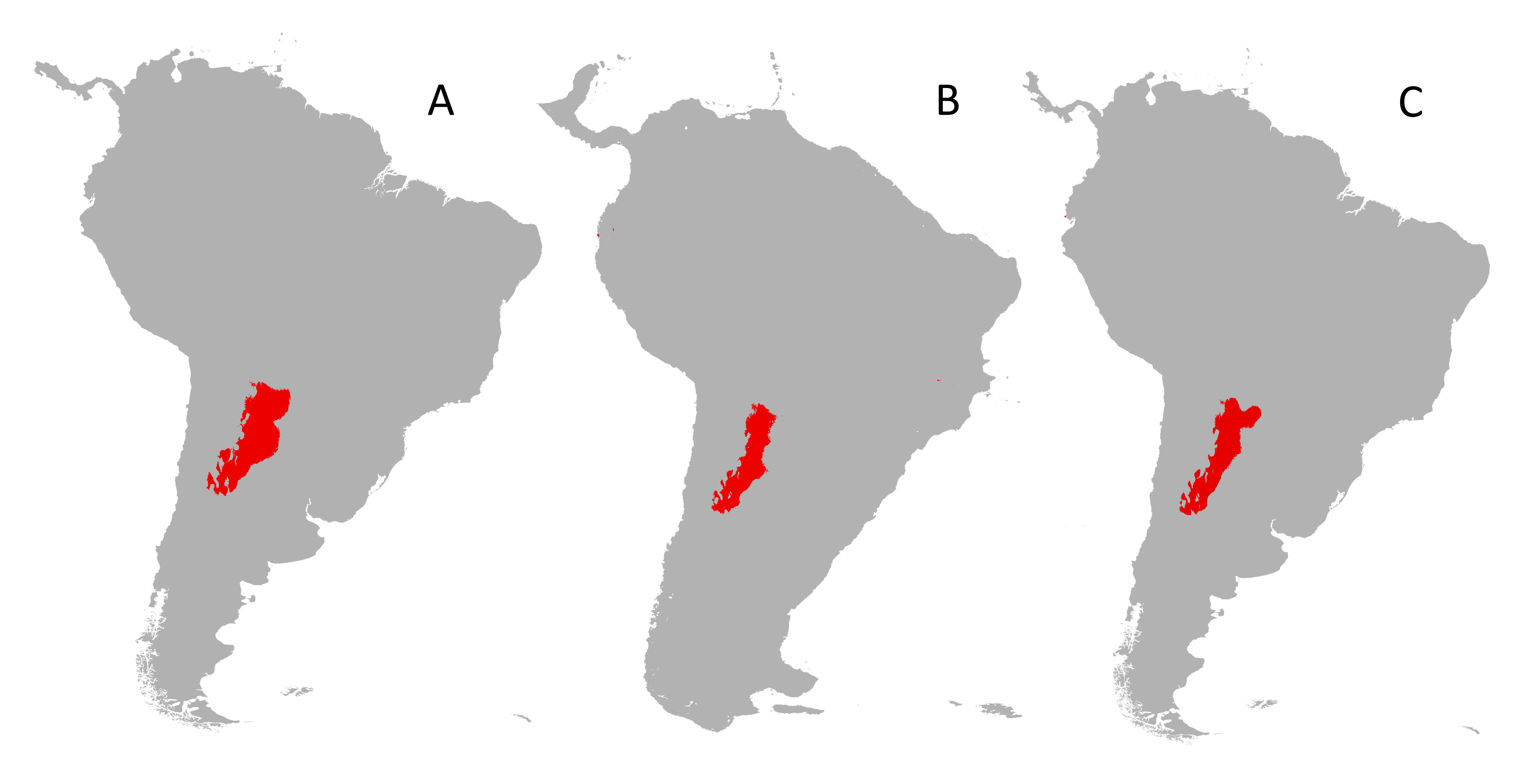

Supplement: S9 Fig — Last interglacial (A); Last glacial maximum (B) and Current (C). (A): 37,905 km2; (B): 31,409 km2 and (C): 31,822 km2. Training data: AUC = 0.992 (A); AUC = 0.991 (B) and AUC = 0.994 (C). Test data: AUC = 0.982 (A); AUC = 0.994 (B) and AUC = 0.940 (C). (TIF) [file pone.0202813.s010.tif]

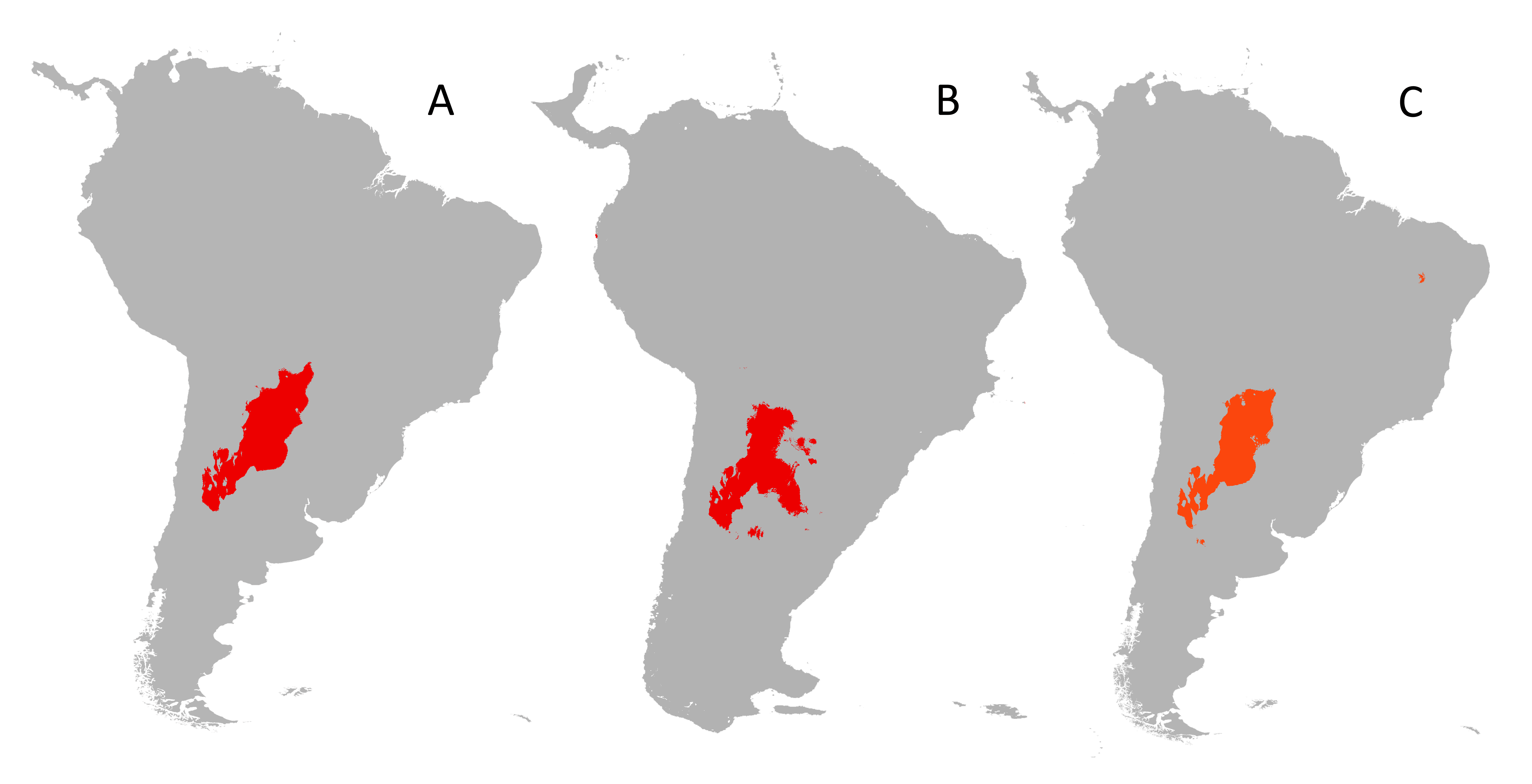

Supplement: S10 Fig — Last interglacial (A); Last glacial maximum (B) and Current (C). (A): 59,958 km2; (B): 74,262 km2 and (C): 55 569 km2. Training data: AUC = 0.979 (A); AUC = 0.980 (B) and AUC = 0.979 (C). Test data: AUC = 0.962 (A); AUC = 0.970 (B) and AUC = 0.954 (C). (TIF) [file pone.0202813.s011.tif]

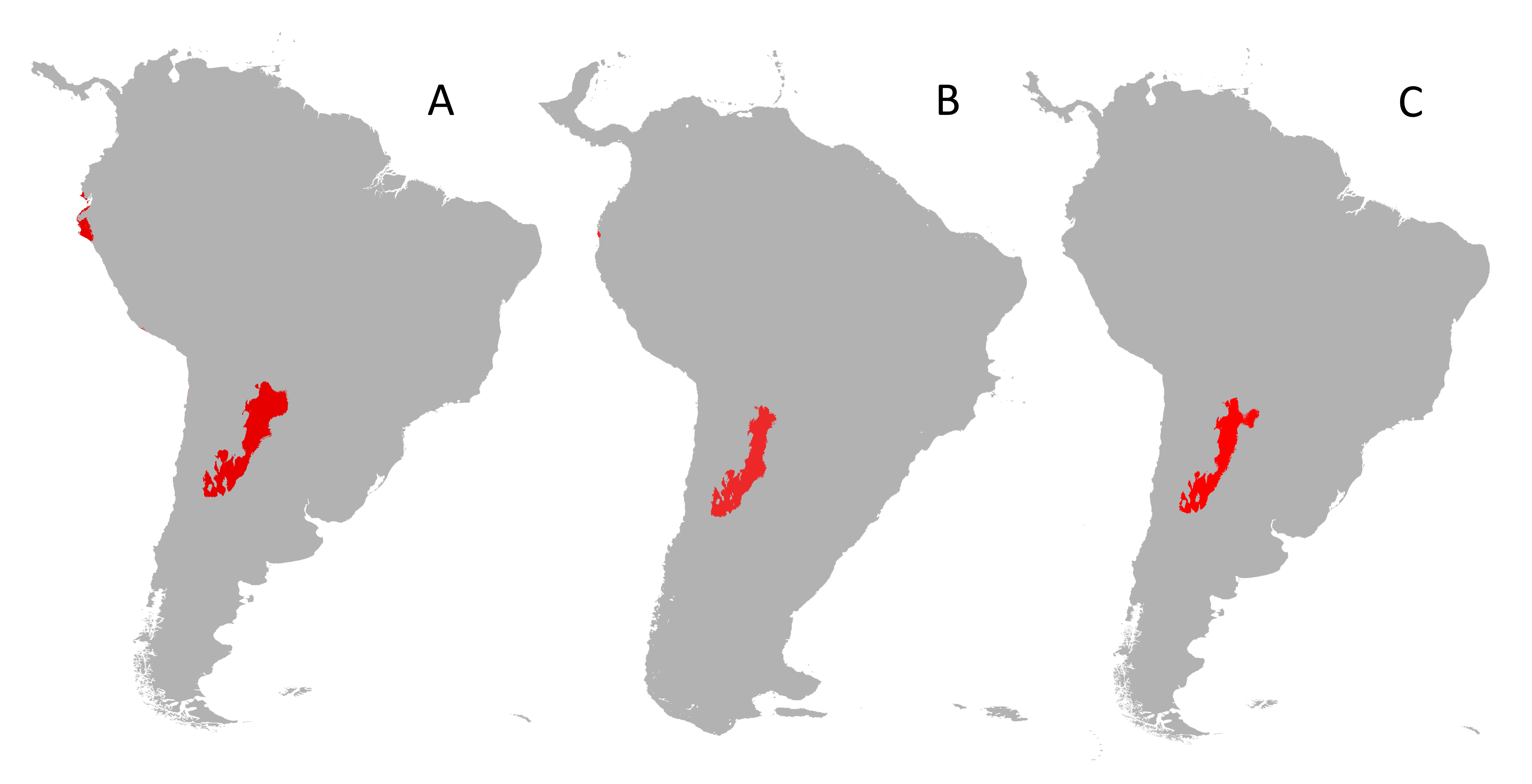

Supplement: S11 Fig — Last interglacial (A); Last glacial maximum (B) and Current (C). (A): 31,170 km2; (B): 29 288 km2 and (C): 24,105 km2. Distribution areas indicated in the western part of the continent are unlikely and represent analysis artifacts resulting from the Grinnellian niche concept. Training data: AUC = 0.994 (A); AUC = 0.989 (B) and AUC = 0.996 (C). Test data: AUC = 0.985 (A); AUC = 0.996 (B) and AUC = 1.000 (C). (TIF) [file pone.0202813.s012.tif]

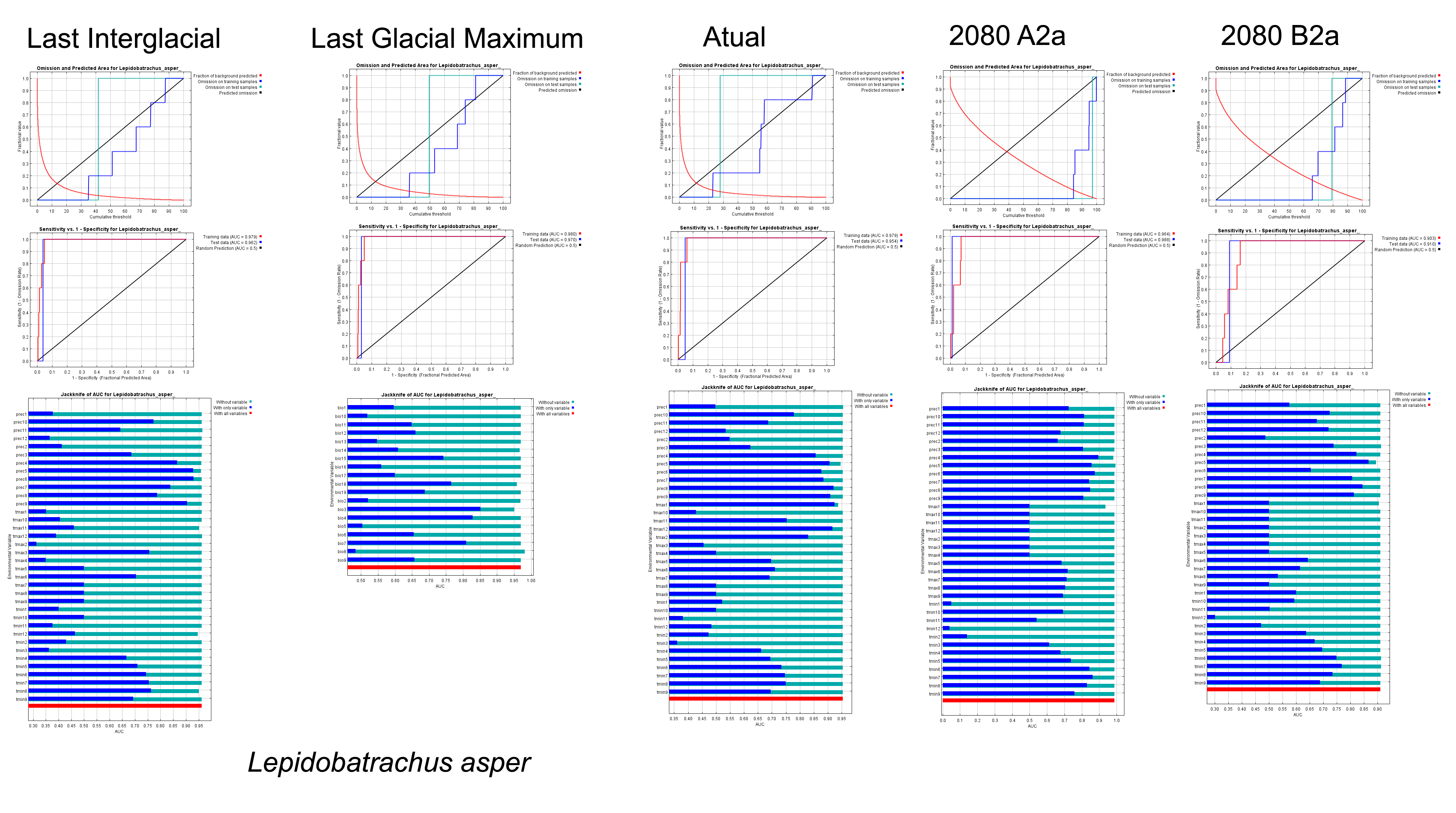

Supplement: S12 Fig — (TIF) [file pone.0202813.s013.tif]

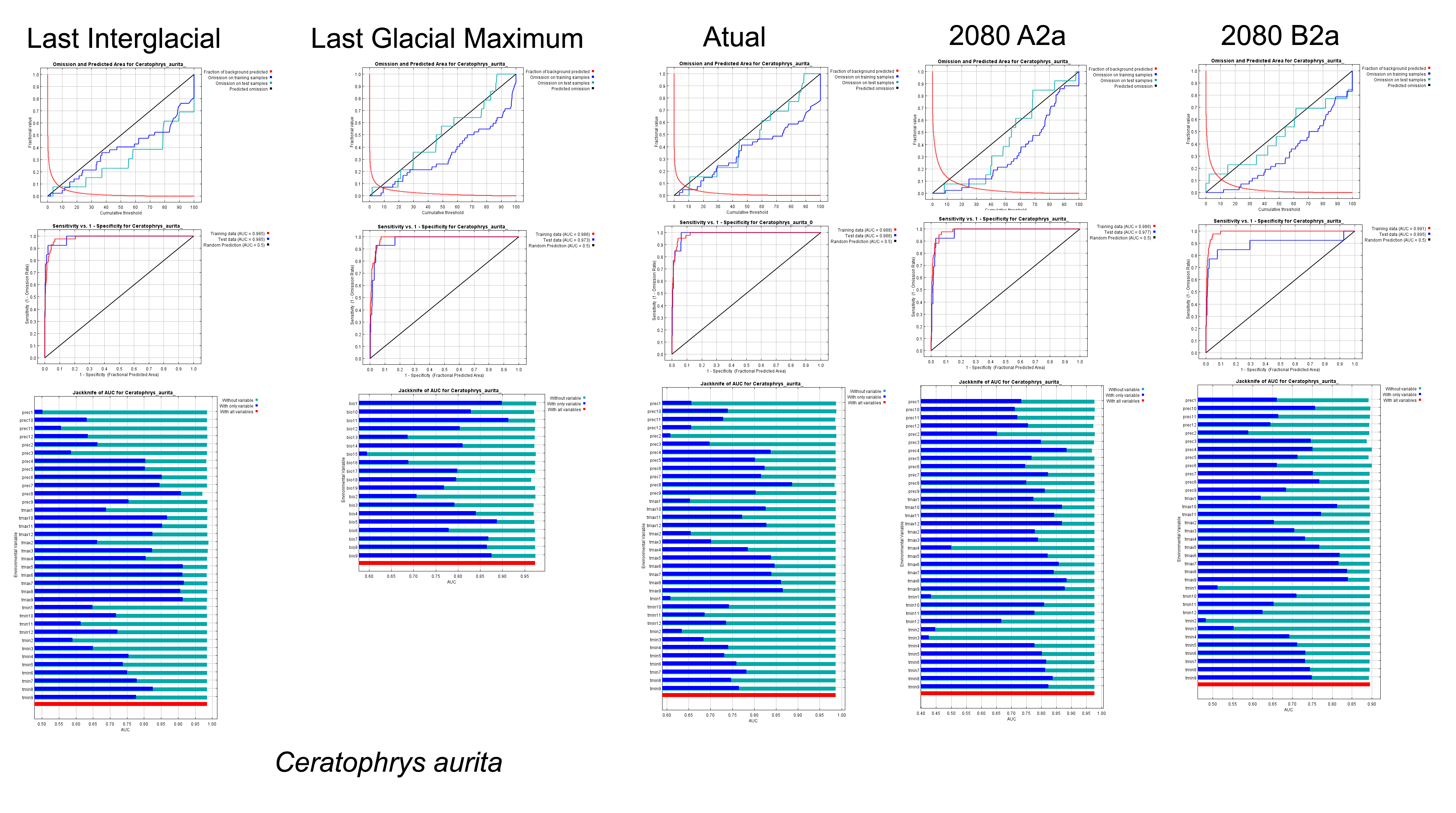

Supplement: S13 Fig — (TIF) [file pone.0202813.s014.tif]

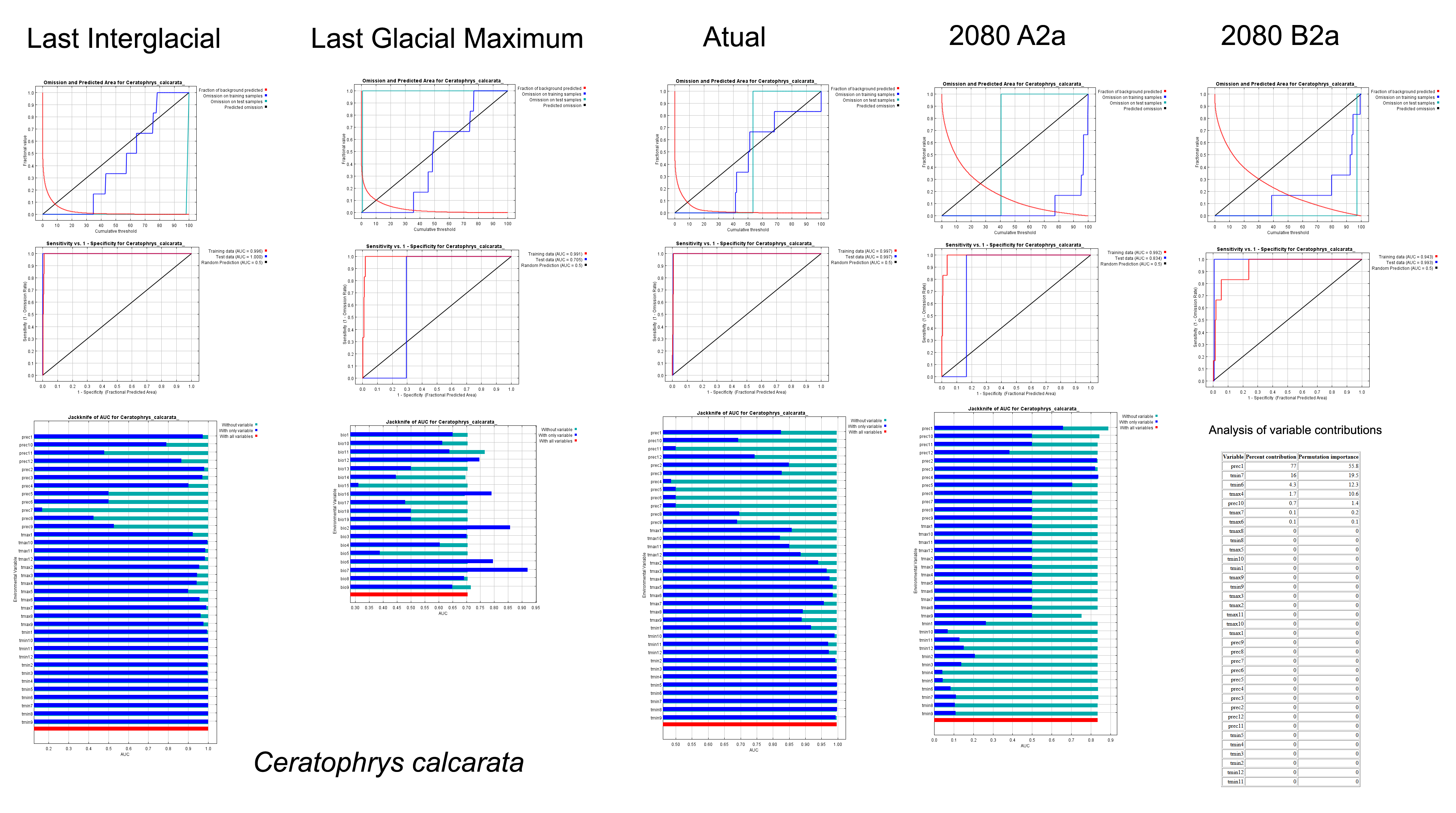

Supplement: S14 Fig — (TIF) [file pone.0202813.s015.tif]

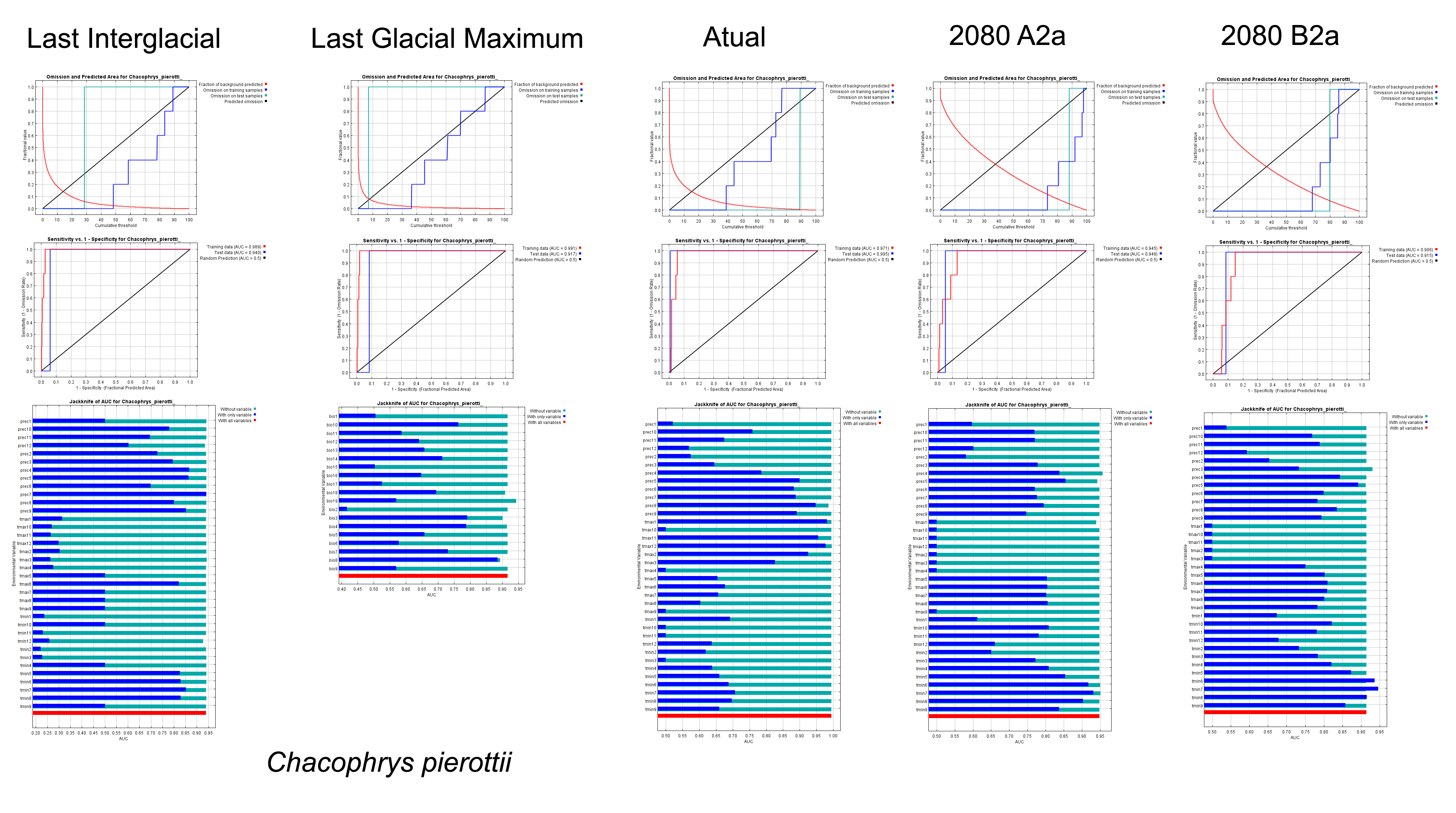

Supplement: S15 Fig — (TIF) [file pone.0202813.s016.tif]

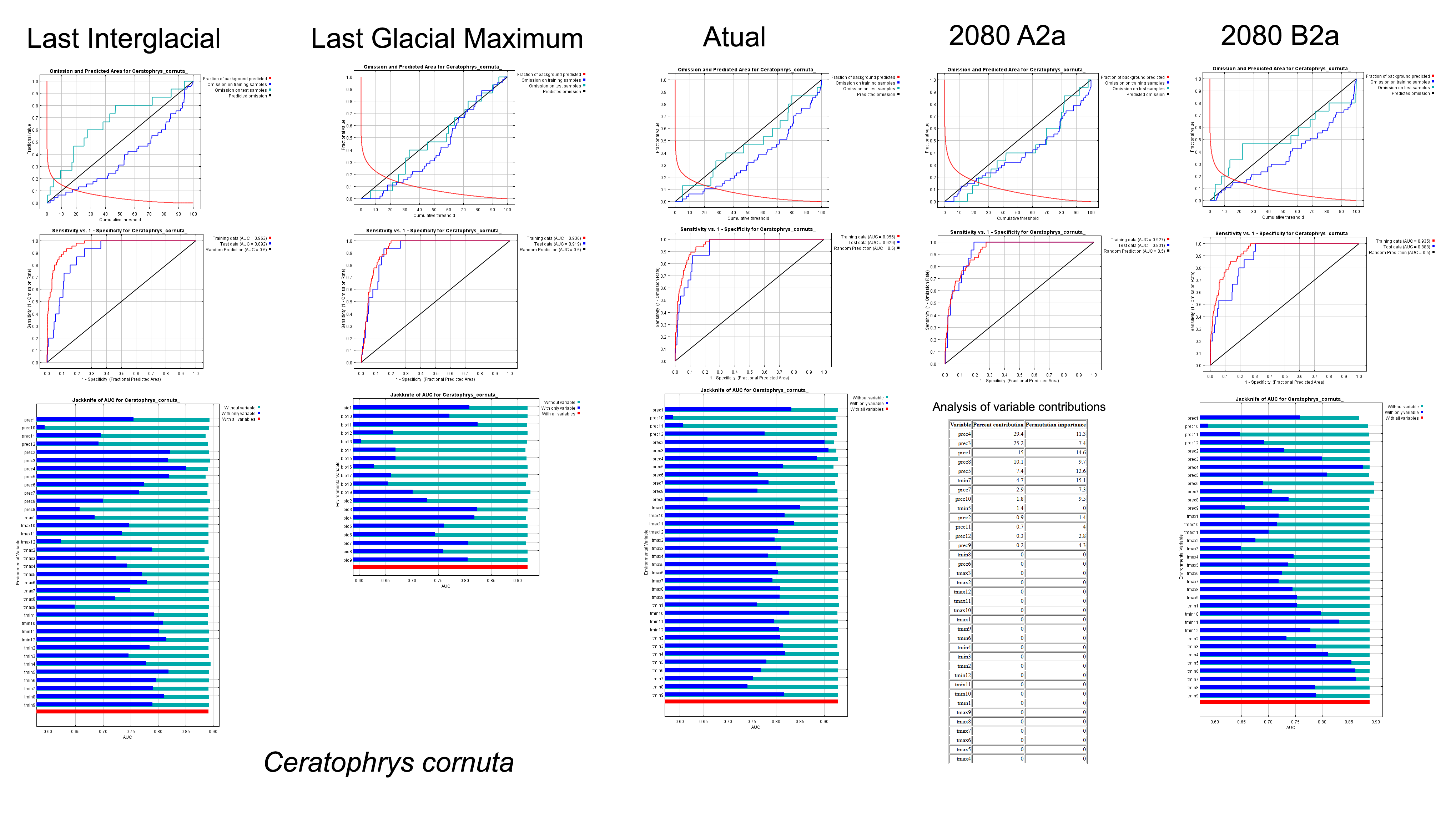

Supplement: S16 Fig — (TIF) [file pone.0202813.s017.tif]

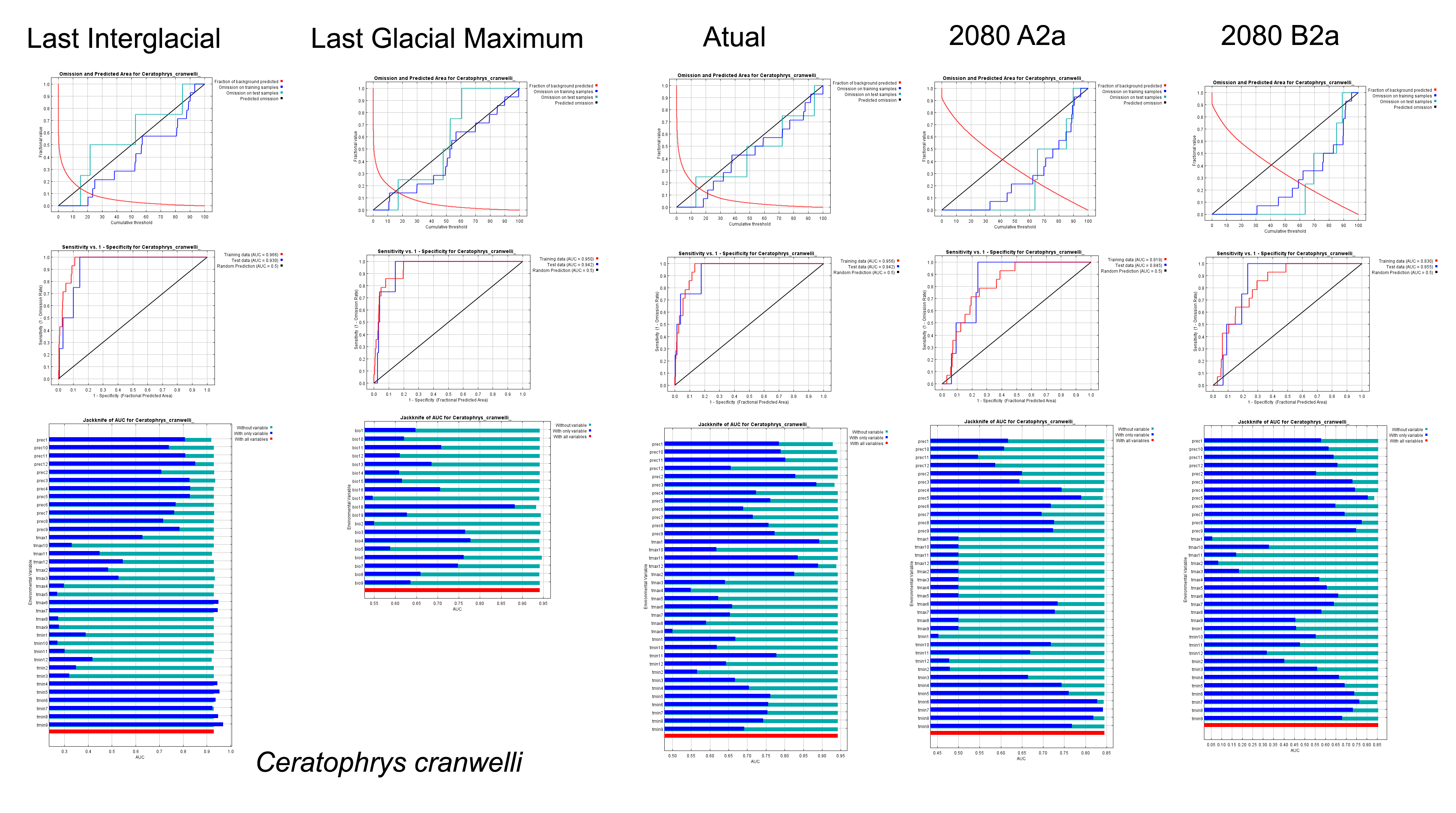

Supplement: S17 Fig — (TIF) [file pone.0202813.s018.tif]

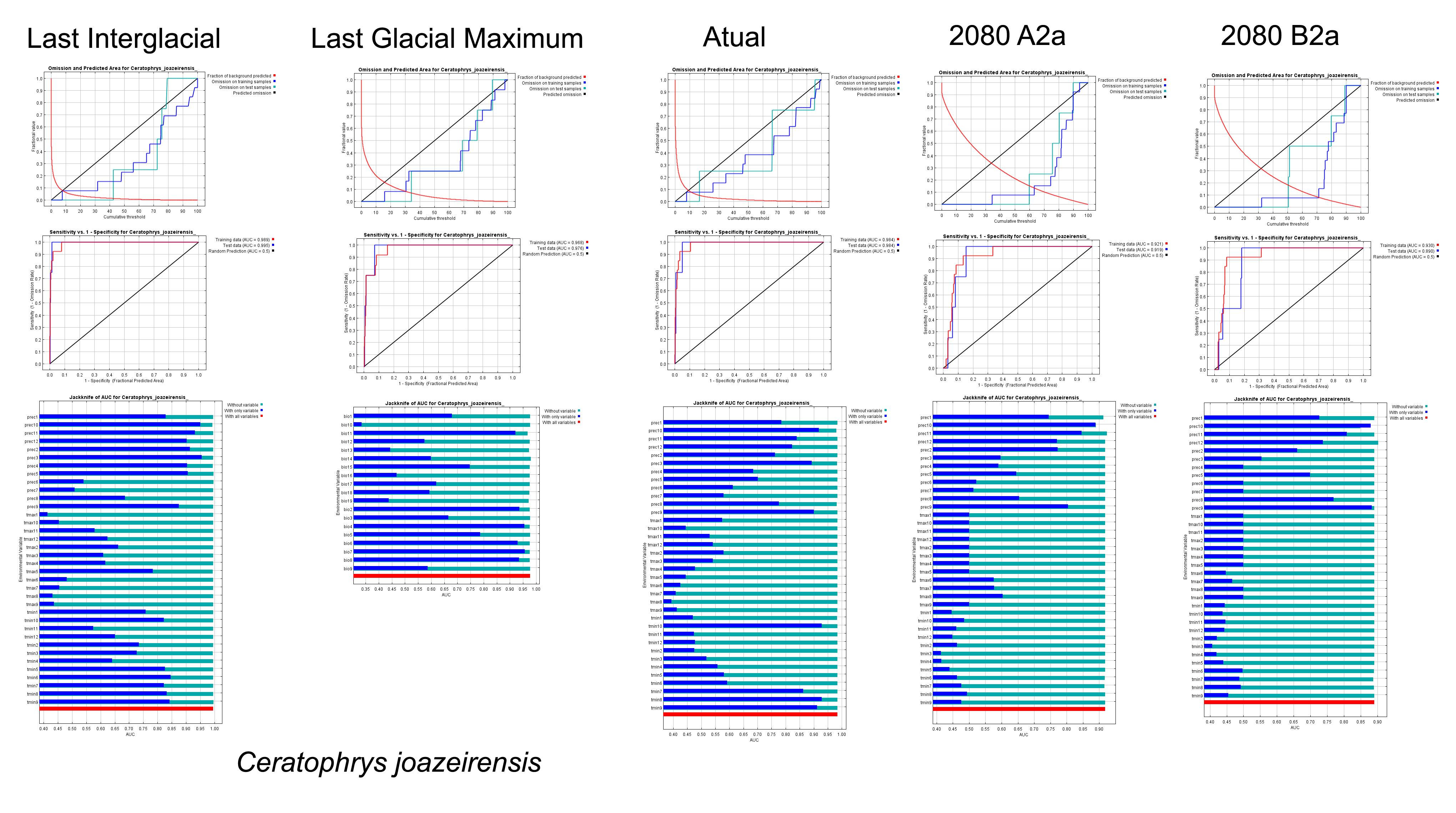

Supplement: S18 Fig — (TIF) [file pone.0202813.s019.tif]

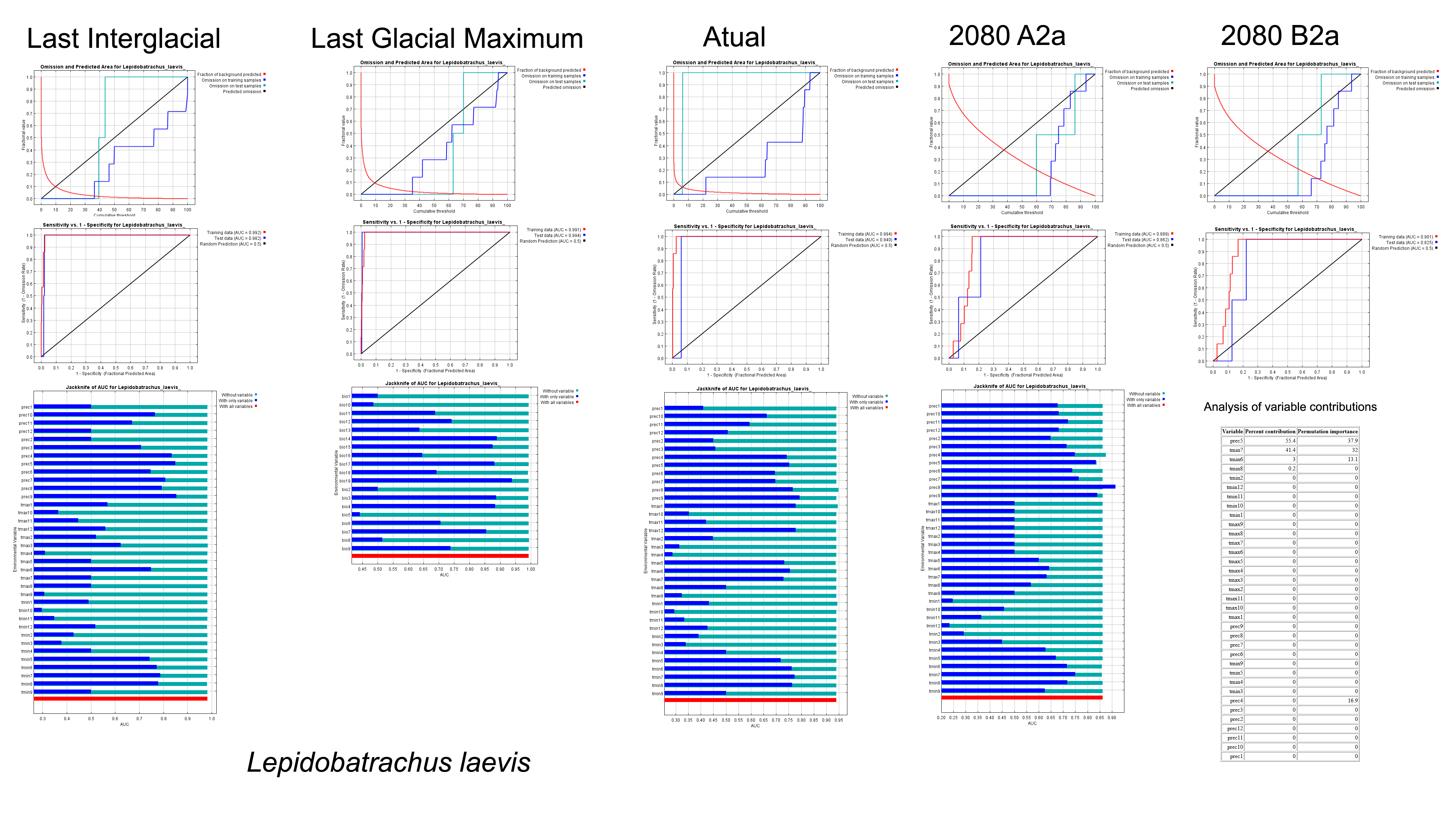

Supplement: S19 Fig — (TIF) [file pone.0202813.s020.tif]

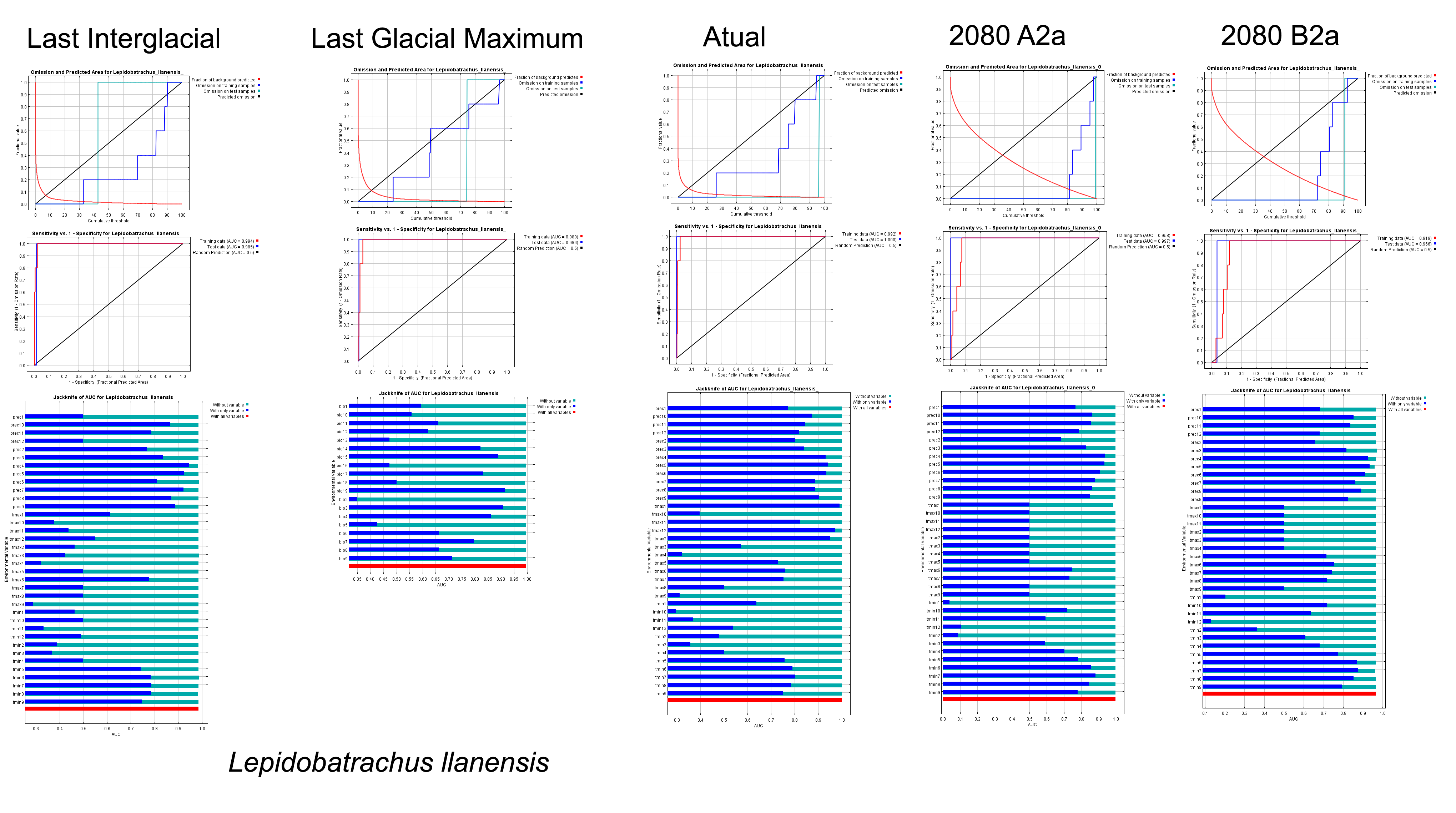

Supplement: S20 Fig — (TIF) [file pone.0202813.s021.tif]

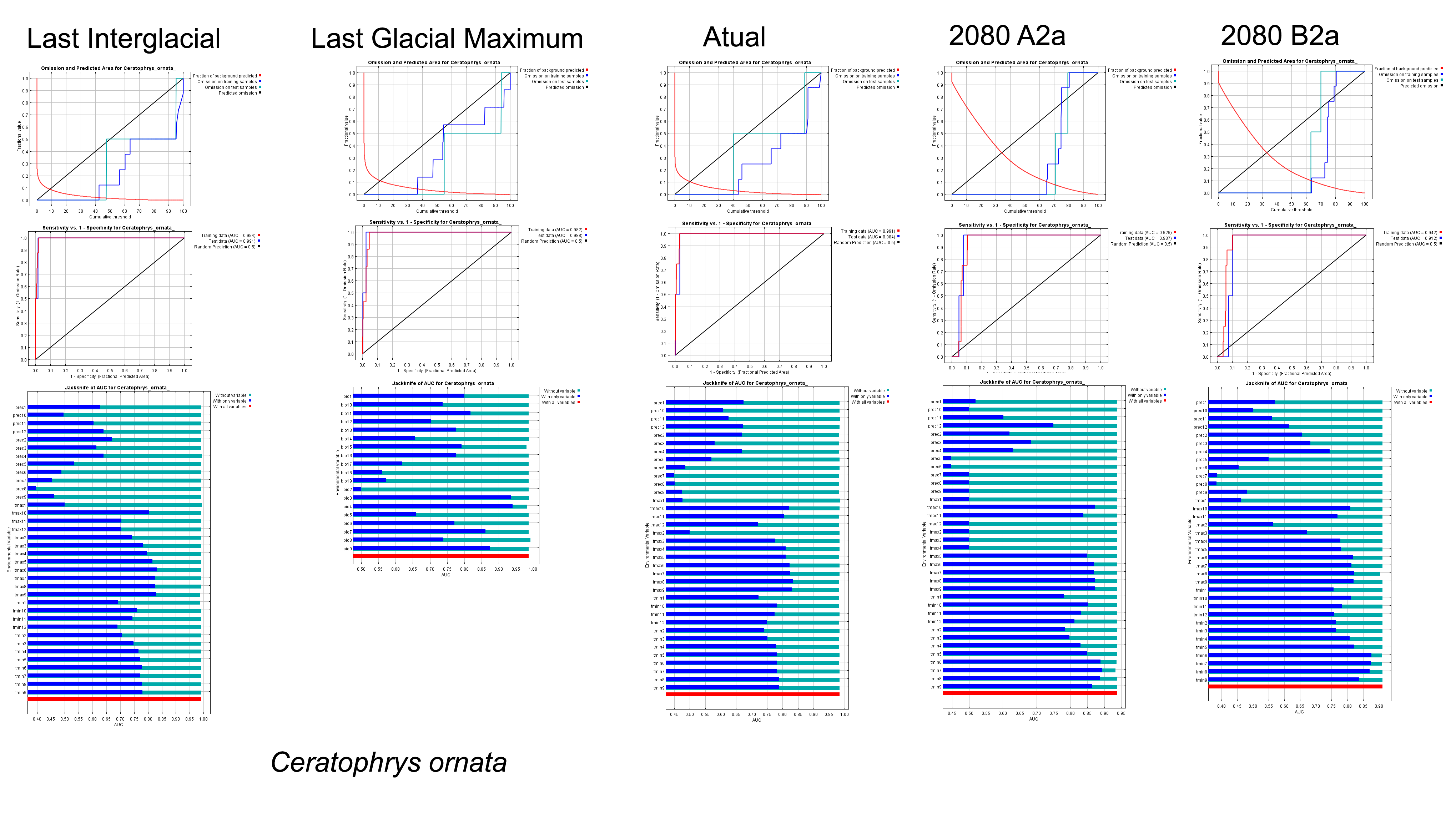

Supplement: S21 Fig — (TIF) [file pone.0202813.s022.tif]

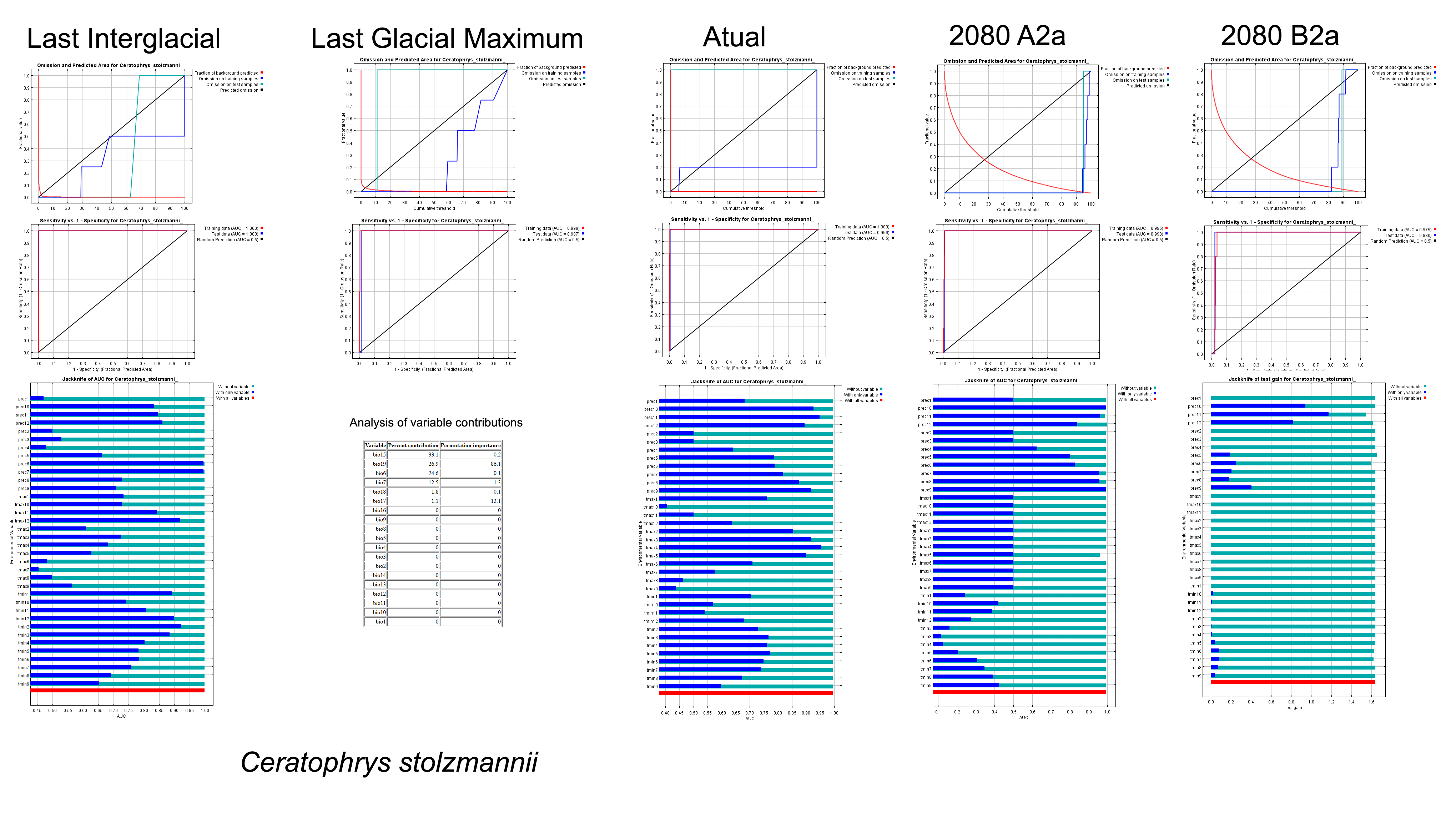

Supplement: S22 Fig — (TIF) [file pone.0202813.s023.tif]
